# Syndrome-oriented CNS infectious complications after haematopoietic stem-cell transplantation and CAR T-cell therapy, part 2—focal brain infections and meningitis of bacterial, fungal, and parasitic origin: recommendations from the EBMT Practice and Harmonization Committee

**DOI:** 10.1016/j.eclinm.2026.104311

**Published:** 2026-11-05

**Authors:** Malgorzata Mikulska, Dina Averbuch, Dionysios Neofytos, Francesco Baccelli, José Luis Piñana, Martin Schmidt-Hieber, Rafael de la Camara, Simone Cesaro, David Navarro, Francesco Onida, Annalisa Ruggeri, Per Ljungman, Isabel Sanchez-Ortega, Ibrahim Yakoub Agha, Jan Styczynski

**Affiliations:** a Division of Infectious Diseases, Department of Health Sciences (DISSAL), University of Genova, Genova, Italy; b Azienda Ospedaliera Metropolitana, IRCCS Policlinico San Martino, Genova, Italy; c Infectious Diseases Working Party, EBMT, Leiden, the Netherlands; d Faculty of Medicine, Hebrew University of Jerusalem, Jerusalem, Israel; e Hadassah Medical Center, Jerusalem, Israel; f Division of Infectious Diseases, University Hospital of Geneva, Geneva, Switzerland; g Pediatric Hematology and Oncology, IRCCS Azienda Ospedaliero-Universitaria di Bologna, Bologna, Italy; h Department of Hematology, Hospital Clínico Universitario of Valencia, Spain; i Med. Clinic 2 (Haematology, Oncology, Pneumology, Nephrology and Diabetology), Medical University Lausitz - Carl Thiem (MUL - CT), Cottbus, Germany; j Hematology División, Hospital de la Princesa, Madrid, Spain; k Pediatric Hematology Oncology, Department of Mother and Child, Azienda Ospedaliera Universitaria Integrata Verona, Italy; l Department of Microbiology, Hospital Clínico Universitario of Valencia, Spain; m Department of Microbiology, School of Medicine, University of Valencia, Valencia, Spain; n Haematology and BMT Centre, ASST Fatebenefratelli-Sacco, University of Milan, Milan, Italy; o Harmonization and Practice Guidelines Committee of the EBMT, Barcelona, Spain; p Haematology and BMT Unit, IRCCS San Raffaele Hospital, Milan, Italy; q Dept. of Cellular Therapy and Allogeneic Stem Cell Transplantation, Comprehensive Cancer Center, Dept. of Infectious Diseases, Karolinska University Hospital, Stockholm, Sweden; r Department of Medicine Huddinge, Karolinska Institutet (KI), Stockholm, Sweden; s EBMT Executive Office, Barcelona, Spain; t CHU de Lille, Univ Lille, INSERM U1286, Infinite, Lille 59000, France; u Department of Pediatric Hematology, Oncology, Immunology and Transplantology, Collegium Medicum, Nicolaus Copernicus University Torun UMK, University Hospital, Bydgoszcz, Poland; v INCLIVA, Biomedical Research Institute, Valencia, Spain; w INCLIVA, Biomedical Research Institute, University of Valencia, Valencia, Spain

**Keywords:** Allogeneic haematopoietic stem cell transplantation, Cellular therapy, CAR T, Invasive fungal infection, Brain abscess, Meningitis, Aspergillosis, Toxoplasmosis, Pneumococcus, Meningococcus, *Listeria*, *Nocardia*

## Abstract

Central nervous system (CNS) infections in patients after haematopoietic stem cell transplantation (HSCT) and CAR T-cell therapy are caused by a broad range of pathogens and associated with high morbidity and mortality. Lack of standardised definitions and uniform diagnostic approach contributes to underdiagnosis and heterogeneity in reporting. This EBMT Practice Harmonization and Guidelines Committee consensus document provides harmonised definition and recommendations on diagnostics, treatment, and response assessment for focal CNS infections and meningitis of bacterial, fungal and parasitic origin in cellular therapy recipients. Focal brain infections are predominantly caused by *Aspergillus* spp, *Toxoplasma gondii*, and *Nocardia* spp, whereas common bacteria are rare. Bacterial or fungal meningitis is rare and typically occurs late post-HSCT. Rapid diagnosis and treatment are crucial to improving outcomes. Benefits of corticosteroids in bacterial meningitis have not been evaluated in this population. Standardised definitions and outcome reporting should be adopted in registries and studies to improve comparability and strengthen the evidence base for management.

## Introduction

Central nervous system (CNS) infections, such as focal parenchymal lesions and meningitis, occurring in immunocompromised patients, and particularly in recipients of cellular therapies such as haematopoietic stem cell transplantation (HSCT) or chimeric antigen receptor (CAR) T-cell therapy, are characterised by a spectrum of causative pathogens and clinical presentations that differ profoundly from those observed in the immunocompetent individuals. Focal brain infection reported in the general population is typically a suppurative parenchymal lesion of infectious origin. While bacteria account for more than over 95% of brain abscesses in immunocompetent patients, fungal or *Toxoplasma gondii* infections predominate among recipients of cellular therapies.^1^, ^2^ Moreover, the incidence of focal parenchymal lesions is significantly higher in immunocompromised patients and is associated with significantly higher mortality than the approximate 10% case-fatality rates reported in the general population.^2^, ^3^, ^4^

Likewise, the incidence of bacterial meningitis, including pneumococcal meningitis, remains significantly higher in immunocompromised hosts compared to the general population, particularly among HSCT recipients. Nevertheless, bacterial meningitis is uncommon among recipients of cellular therapies compared with other CNS infections, with viral encephalitis occurring more frequently, followed by focal parenchymal lesions of fungal or parasitic origin.^4^, ^5^ Finally, clinical manifestations and radiological findings are often markedly non-specific in the setting of immune dysfunction, as many reflect the host immune response to the pathogen rather than the pathogen itself, thereby further hindering accurate and timely diagnosis.^6^, ^7^

The aim of this document, developed by the EBMT Infectious Diseases Working Party (IDWP) and the Practice Harmonization and Guidelines Committee (PH&G) is to provide insights into the definitions, epidemiology, differential diagnosis, diagnostic algorithms and management of focal parenchymal brain lesions and meningitis of bacterial, fungal and parasitic origin in recipients of HSCT or CAR T-cell therapy. This overview is intended to facilitate reporting of CNS complications in this population for research and registry purposes (diagnostic and outcome definitions) and to harmonise best practice clinical management (diagnostic workup algorithm and empirical therapy). Viral infections are addressed in the companion manuscript.

## Methods

### Search strategy and selection criteria

This manuscript constitutes Part 2 of a two-part EBMT expert-based practice harmonisation project. Both companion manuscripts were developed through the structured literature review and multidisciplinary expert consensus process.^8^ This paper focuses on focal brain lesions and meningitis of bacterial, fungal or parasitic origin, while viral meningitis is covered in Paper 1.

Before the workshop, predefined clinical questions were formulated, and a structured literature review of the published registry analyses, cohort studies, case series, and systematic reviews was performed. A comprehensive literature search was conducted in PubMed to identify relevant studies published between January 1, 2000 and September 2025. The search strategy combined Medical Subject Headings (MeSH) terms and free-text keywords related to central nervous system (CNS) infections and haematopoietic transplantation. Detailed literature search protocols are reported in Supplementary Material, page 3.

## EBMT workshop and consensus

A multidisciplinary panel of haematology (n = 4), infectious diseases (n = 3), microbiology (n = 1), paediatric specialists (n = 4), and members of the EBMT PH&G Committee (n = 4) was convened. Committee members were selected and invited based on their clinical and scientific expertise in the field and the involvement in dedicated IDWP subcommittees, with consideration given to geographic diversity and representation of complementary specialties and competencies.

Designated panel members reviewed and summarised the evidence relevant to each question and circulated it to the group. Panel members discussed the evidence and proposed recommendations during a two-day EBMT workshop. Available evidence and preliminary recommendations were presented and discussed in plenary sessions. After the workshop, the two companion manuscripts were circulated to the panel members.

Given the scarcity and heterogeneity of the available evidence in the targeted patients’ population, particularly the absence of randomised trials or comparative observational studies for most diagnostic and therapeutic interventions, and the frequent need to extrapolate from other populations, we elected not to formally grade the evidence or the strength of recommendations. Instead, the supporting evidence and its limitations were described narratively. Recommendations based predominantly on indirect evidence, good clinical practice, or multidisciplinary expert consensus were explicitly formulated, and their wording was adjusted to reflect the associated uncertainty. Consensus was reached through iterative discussion and review of the relevant literature, with disagreements addressed and recommendations revised until all panel members were in agreement. The final recommendations were unanimously approved by the panel before internal review and validation in accordance with the EBMT PH&G Committee process in 2025. The EBMT performed administrative and legal management, as well as funding acquisition. No pharmaceutical company and no other funding source were involved in any way or had a role in the design, data collection, data analysis, data interpretation, or writing of the report.

## Epidemiology and risk factors

In the identified studies (Table 1), bacterial or fungal CNS infections were reported in 0–2% of included patients, while the rate of *T. gondii* CNS infections was usually <1% (range 0–1.8) in studies published after 2000.^4^, ^10^, ^12^, ^13^, ^14^, ^15^, ^16^, ^17^, ^18^, ^19^, ^20^, ^21^, ^22^, ^23^, ^24^ In a recent multicentre prospective European study, the rate of CNS infections, other than viral, was even lower (<0.5%).^9^ Details from studies on bacterial, fungal and parasitic infections in HSCT recipients are reported in Supplementary Tables S1–S5. Most CNS infections were reported in allogeneic HSCT recipients, with few cases in autologous HSCT recipients, and even fewer after CAR T cells. Therefore, most data and recommendations are relevant mainly for allogeneic HSCT population.

**Table 1:** Pathogen-specific burden of CNS infections after HSCT details from cohort studies that reported the rates of any CNS infection after HSCT, and CNS infection due to specific pathogens: bacterial, fungal and *Toxoplasma*.

Study: first author and year of publication (country, population if paediatric only)	Total HSCT recipients, n (type of HSCT)	HSCT recipients with CNS infection, n	Number of infections due to specific pathogens; Rate of CNS infections among all HSCT recipients^a^		
			Bacterial	Fungal	*Toxoplasma gondii*
Prospective					
Schmidt-Hieber 2026 (Multicentre EBMT)^9^	2910 (1735 allo-HSCT, 1175 auto-HSCT)	21 all after allo-HSCT	2; 0.07% for any HSCT, 0.11% for allo-HSCT; (10%)	3 (all IA); 0.1% for any HSCT, 0.17% for allo-HSCT; (14%)	5; 0.17% for any HSCT, 0.28% for allo-HSCT; (24%)
Schmidt-Hieber 2020 (Multicentre EBMT)^4^	4063 (2136 allo-HSCT, 1927 auto-HSCT)	58 for any HSCT, 56 after allo-HSCT, 2 after auto-HSCT	5; 0.12% for any HSCT, 0.23% for allo-HSCT; (9%)	23; 0.57% for any HSCT, 1.1% for allo-HSCT; (40%)	10; 0.25% for any HSCT, 0.42% for allo-HSCT; 0.05 for auto-HSCT; (17%)
Retrospective					
Zaidman 2022 (Israel; paediatric)^10^	746 (allo-HSCT)	28	7; 0.94%; (25%)	3: 0.40%; (11%)	8; 1.07%; (29%)
Mannina 2020 (Germany)^11^	888 (allo-HSCT)	9	0	0	1; 0,11%; (11%)
Dowling 2018 (USA)^12^	263 (allo-HSCT)	3	1; 0.38%; (33%)	0	1; 0.38%; (33%)
Hanajiri 2017 (Japan)^13^	353 (allo-HSCT)	17	7; 1.98%; (41%)	0	1; 0.28%; (6%)
Balaguer-Roselló 2017 (Spain)^14^	709 (allo-HSCT)	34	4 (0.56%; 12%)	12 (1.7%; 35%)	4 (0.56%; 12%)
Colombo 2017 (Italy)^15^	452 (allo-HSCT)	9	2; 0.4%; (22%)	3; 0.7%; (33%)	2; 0.4%; (22%)
Barba 2009 (Spain)^16^	191 (allo-HSCT)	6	0	1 (0.5%; 17%)	0
Weber 2008 (Germany, paediatric)^17^	165 (111 allo-HSCT, 54 auto-HSCT)	9, bacterial, fungal and parasitic infections only after allo-HSCT	0	2; 1.2% for any HSCT, 1.8% for allo-HSCT; (22%)	3; 1.8% for any HSCT, 2.7% for allo-HSCT; (33%)
Denier 2006 (France)^18^	361	15	1; 0.28%; (7%)	2; 0.55%; (13%)	5; 1.4%; (33%)
Faraci 2002 (Italy, paediatric)^19^	272 (185 allo-HSCT, 87 auto-HSCT)	7, bacterial, fungal and parasitic infections only after allo-HSCT	0	1; 0.4% for any HSCT, 0.5% for allo-HSCT; (14%)	1; 0.4% for any HSCT, 0.5% for allo-HSCT; (14%)

Allo, allogeneic; auto, autologous; CNS, central nervous system; HSCT, haematopoietic stem cell transplant; IA, invasive aspergillosis.

a In brackets rates of CNS infections due to bacteria, fungi or *Toxoplasma gondii* among all reported CNS infections, the remaining % value refers to CNS infections of viral origin.

### Focal brain infection

Most of the reported brain parenchymal lesions in cellular therapy recipients were caused by fungi (mainly *Aspergillus* spp) and *T. gondii*, in both adult and paediatric cohorts, while there are very limited data on bacterial abscesses (Table 1, Supplementary Tables S1–S4). In the studies that specifically reported on the focal brain lesion rate, brain abscess after HSCT was consistently rare (below 2%).^4^, ^14^ However, in an autopsy series in HSCT recipients, the focal brain infection rate was particularly high (13%), due to predominantly fungal pathogens,^23^ while in other studies *T. gondii* infection predominated^10^, ^18^, ^20^ and some recent data showed a higher rate of bacterial aetiology compared to older studies.^10^, ^13^

Whereas in the general patient population *Streptococcus* spp, *Staphylococcus* spp and Enterobacterales are the most frequent pathogens,^2^ they are almost absent in the HSCT setting, while nocardiosis is one of the most frequently identified CNS infections of bacterial origin.^25^, ^26^, ^27^ The rarity of bacterial abscesses in HSCT recipients may be due to, in part, the high frequency of empirical antibiotic therapy, prolonged antibiotic courses, and the administration of antibacterial prophylaxis. Despite being the main cause of focal brain infections after HSCT, CNS invasive fungal infections (IFI) after HSCT are rare (<1%; Supplementary Table S3).^14^, ^28^, ^29^, ^30^, ^31^, ^32^, ^33^, ^34^ CNS IFI occurred at a median onset of 5–6 months post-HSCT and in most studies at least one extra-CNS infection site, predominately lungs (50–96.6% of cases), was identified a median 5 weeks before the CNS IFI diagnosis. All patients with CNS IFI due to moulds presented with focal brain lesions. *Aspergillus* spp remains the most identified pathogen, followed by Mucorales, and non-*Aspergillus* hyaline moulds, such as *Fusarium* spp and *Scedosporium* spp Cryptococcal meningitis was the only fungal meningitis described. Overall mortality was up to 100% in the studies published prior to 2017, while among studies published after 2017, 30 to 84-day mortality was in the range of 34.5%–51.8%, with overall mortality up to 92% (Supplementary Table S3). This may be partly attributed to earlier diagnosis and prompt initiation of effective treatment. The risk of CNS IFI in patients receiving CAR T-cell therapy has not been clearly defined, with only a few cases described.^35^

Parasitic CNS infections after HSCT are mainly CNS toxoplasmosis, while only a few reports of non-*T. gondii* infections after HSCT have been published. Details of studies on parasitic CNS infections are in Supplementary Material, including Supplementary Tables S4 and S5.

### Meningitis

The rate of infectious meningitis after cellular therapy is rarely reported as a dedicated endpoint, and meningitis is often grouped within broader categories such as CNS infections, infectious neurological complications, or meningoencephalitis. Consequently, the true epidemiological burden of meningitis after HSCT remains difficult to quantify, and comparisons across studies are limited by heterogeneous definitions and diagnostic criteria. Few single-centre adult studies specifically address bacterial meningitis, reporting a generally low incidence ranging from 0.3% to 0.6% across different HSCT cohorts.^12^, ^14^, ^15^, ^36^ In a Brazilian study, meningitis accounted for approximately 3% of all neurological complications after HSCT.^21^ Umbilical cord blood transplantation has been associated with a higher incidence of CNS infections, including bacterial, likely related to delayed immune reconstitution, higher rates of graft failure or delayed engraftment, and prolonged immunodeficiency.^12^, ^14^ In the Japanese study reporting mainly pre-engraftment meningitis, all cases were associated with prior or concomitant bacteraemia with the same bacteria.^36^

The risk of late, community-acquired bacterial meningitis in HSCT was reported in a population-based prospective study as over 50 times higher in allogeneic HSCT recipients and over 10 times higher in autologous HSCT recipients compared to the general population.^37^ Most episodes occurred late after HSCT, in median 4 years after HSCT. A classical triad of fever, neck stiffness and change in mental status was present in 36% of HSCT patients, somewhat lower than in the general population.^38^ HSCT recipients and other onco-haematological patients with meningitis more often presented with low CSF leucocyte counts. CT scan abnormalities were identified in less than 50% of cases and were compatible with sinusitis in all of them.^37^ The rate and aetiology of bacterial meningitis are summarised in Supplementary Table S2. The proportion of bacterial causes among CNS infections after HSCT was highly variable, likely reflecting differences in definitions across studies, with most studies reporting rates ranging from 5 to 10% of all CNS infections. A review of 18 bacterial meningitis cases after HSCT reported *Listeria monocytogenes* being the most frequent pathogen (7, 39%), followed by *Streptococcus pneumoniae* (3, 17%) and *Enterococcus faecium* (3, 17%) cases, and single cases of *Staphylococcus aureus*, *Bacillus cereus*, *Elizabethkingia meningoseptica* and *Stomatococcus mucilaginosus*.^37^ Single meningitis cases due to *S. aureus*,^39^, ^40^ or *B. cereus* were reported in HSCT recipients.^41^, ^42^ Despite a significantly higher incidence of *S. pneumoniae* infections after HSCT, in studies on invasive pneumococcal disease in these patients, CNS involvement was very rare (<5%) or even absent in many (Supplementary Table S2). In the aforementioned prospective study documenting higher risk of community acquired meningitis in HSCT recipients over eight years, among 14 cases reported, 11 were caused by *S. pneumoniae* and two by *Neisseria meningitidis*.^37^ Details on *L. monocytogenes* and *Mycobacterium tuberculosis* meningitis are reported in Supplementary Material.

## Harmonised definitions

### Purpose and scope

The following harmonised definitions were established to enable uniform case classification, reporting, and outcome assessment of focal brain infection or meningitis in recipients of HSCT or CAR T-cell therapy (Table 2). Each definition is structured to allow grading of diagnostic certainty (proven or probable) and to incorporate objective parameters for assessing treatment response, thereby supporting either diagnostic, clinical management or research comparability. Data on CNS infections after CAR T-cell therapy are limited, but there are several shared risk factors with HSCT; thus, diagnostic and management workup is supported by data from the HSCT population.

**Table 2:** Definitions of CNS infection due to bacterial, fungal, or parasitic agents in recipients of cellular therapy. Definitions do not include viral pathogens which are addressed in the companion paper.

Entity	Focal brain lesion of infectious origin	Meningitis
Definitions	Focal CNS infection is defined based on the presence of radiological image of brain lesion(s) on imaging (CT or, preferably MRI) and evidence of proven or probable infectious origin	Infectious meningitis is defined based on the presence of meningitis, defined as relevant clinical presentation and evidence of meningeal inflammation^b^ and proven or probable infectious origin
Relevant clinical presentation	Symptoms may vary from absent to severe: fever and/or headache, decreased consciousness and focal symptoms based on localisation, including seizures and focal neurological signs, e.g., plegia or paresis, ataxia, aphasia, seizures, visual field loss, sensory loss, etc.	Symptoms may vary from mild to severe and include the following: fever and/or signs and symptoms of CNS disorder, such as vomiting, headache, photophobia, irritability, confusion, mental status changes, seizures, lethargy, nuchal rigidity, or other signs of meningeal irritation. In infants, it can include bulging fontanelle, irritability, poor feeding, respiratory distress, pale or marble skin and hyper- or hypotonia
Proven infectious origin	Proven focal brain infection is defined as brain lesion(s) with the identification of a bacterial, fungal or parasitic pathogen in the brain tissue obtained by aspiration or at autopsy with histopathological exam, direct microscopy or culture or in the CSF by positive cryptococcal antigen or culture for fungi, *Nocardia* spp., or mycobacteria. A positive molecular test (PCR, NGS) on pathological brain tissue can identify the suspected fungus, *T. gondii*, or mycobacteria	Proven infectious meningitis is defined as presence of meningitis together with the identification of a plausible bacterial, fungal, or parasitic pathogen in the CSF by direct microscopy, culture, or cryptococcal antigen test
Probable infectious origin	Probable focal brain infection due to a bacterial, fungal, or parasitic pathogen is defined as brain lesion(s) with one of the following and when other aetiology is reasonably excluded: • In CSF (one of the following): ○ Positive culture for a plausible bacterial pathogen, other than *Nocardia* spp. and mycobacteria (see criteria for proven) ○ Positive molecular test (PCR, NGS) for bacteria^c^, *T. gondii*, *Nocardia* spp. or fungi^d^^a^ ○ Positive fungal antigens (GM, BDG)^e^ • In other specimens (blood, BAL): culture or molecular test (PCR, NGS) for a pathogen with high likelihood of dissemination to CNS (e.g., *Nocardia* spp., *Listeria monocytogenes*, *M. tuberculosis*, filamentous fungi, *T. gondii*) or GM^e^	Probable infectious meningitis is defined as presence of meningitis and a plausible infectious pathogen identified with one of the following when CSF before initiation of antimicrobials was not obtained and when other aetiology is reasonably excluded: • In CSF: evidence of a positive molecular test^b^, such as PCR or NGS, or pneumococcal antigen • In blood: positive blood culture for a pathogen typically associated with meningitis, such as *Streptococcus pneumoniae, Haemophilus influenzae, Neisseria meningitidis, Listeria monocytogenes*

BAL, bronchoalveolar lavage; BDG, beta-D-glucan; CNS, central nervous system; CSF, cerebrospinal fluid; CT, computed tomography, GM, galactomannan; MRI, magnetic resonance imaging, NGS, next generation sequencing; PCR, polymerase chain reaction.

a For harmonization purposes we can define such cases as probable fungal CNS infection even though some are not present in IFI criteria.^43^

b Meningeal inflammation is defined as presence of at least one of the following: (a) abnormal CSF analysis with pleocytosis (>5 white cells/μLin adults or above age-related norms in children); abnormal CSF analysis can include increased protein and decreased glucose and/or (b) meningeal enhancement compatible with meningitis on contrast enhanced brain CT or MRI (if lumbar puncture was not performed recently as lumbar puncture can cause a non-specific meningeal enhancement). In severely immunocompromised patients (e.g., profound neutropenia <100 cells/mm^3^), the signs of inflammation (pleocytosis or imaging finding) can be attenuated. Case-by-case discussion is needed when the patient with suspected meningitis and identification of the plausible pathogen in the non-bloody CSF presents with a complete absence of meningeal inflammation.

c Molecular tests, such PCR and NGS, are very sensitive, but variable rates of false positive results have been reported even for typical pathogens, particularly in case of multiplex platforms, moreover interpretation of positive results for pathogens that are rare or atypical for CNS infections require careful evaluation, especially in severely immunocompromised patients, and warrants further studies.^44^, ^45^

d For harmonization purposes we consider such cases as probable fungal CNS infection even though some are not present in the 2019 revised definition guidelines.^46^

e Cut-off for positivity will depend on the assay used. For GM Platelia *Aspergillus* (Bio-Rad) assay, optical density index (ODI) cut off 1 is recommended for higher specificity, but exposure to mould-active antifungals might reduce its sensitivity.^46^

### Focal brain infection

A focal brain infection, also reported as a brain abscess, is classically defined in the general population as a focal parenchymal, usually suppurative, brain lesion of infectious origin which may start as localised cerebritis and subsequently convert into a pus collection within a well-vascularised capsule.^1^, ^47^ However, these characteristics might be absent in immunocompromised hosts, both due to immune deficits, including neutropenia, and the nature of typical pathogens (e.g., *T. gondii* or fungi), less frequently associated with a marked suppurative reaction characterising pyogenic bacteria. Highlighting these specific features, focal brain infection referring to either single or multiple lesions, appears to be a more fitting term than brain abscess in patients after cellular therapy. Although definitions of proven and probable fungal and *T. gondii* infections in immunocompromised patients have been previously developed, certain limitations should be acknowledged.^46^, ^48^ First, they do not provide definitions for probable CNS infections. For instance, it is unclear if positive fungal PCR or antigens or *T. gondii* PCR in blood in patients with brain lesions are sufficient to define a probable fungal or parasitic CNS infection, respectively. Secondly, molecular results, such as PCR or NGS (either 16S/18S sequencing or mNGS), were not included. Therefore, our proposed definitions are in agreement with the existing ones for proven focal brain infections,^46^, ^48^ while specific considerations for cellular therapy patients were included for the definition of probable focal infections (Table 2). Briefly, proven focal brain infections are defined as radiologically confirmed lesion(s) with documented infectious origin by identification of bacterial, fungal or parasitic pathogen by histopathological exam, direct microscopy, or culture on a neurosurgically (or by autopsy) obtained sterile CNS sample (biopsy, aspiration, excision biopsy), and in rare cases in CSF.^46^, ^48^ For probable CNS infections, documentation of probable infectious aetiology can be obtained either in the CSF or other specimens (e.g., blood or BAL), for certain pathogens typical for cellular therapy recipients, with high likelihood of dissemination to the CNS, and if no other plausible cause is detected.

### Meningitis

International guidelines define meningitis in the general population as inflammation of the meninges, causing severe disease.^43^, ^49^ British guidelines define meningeal inflammation as CSF pleocytosis, and increased protein levels associated with meningeal contrast enhancement on CT scan or MRI.^50^

Table 2 presents our proposed definition of meningitis in patients undergoing cellular therapy. Briefly, we propose to define infectious meningitis based on the presence of relevant symptoms, documentation of proven or probable infectious aetiology and the evidence of meningeal inflammation (either CSF pleocytosis or meningeal enhancement on contrast-enhanced brain CT scan or MRI) (Table 2).^37^, ^51^ Decreased inflammatory response in severely immunocompromised patients may make the meningitis diagnosis challenging, as clinical presentation can be non-specific and CSF pleocytosis and the imaging features of meningeal inflammation may be subtle.^38^

## Harmonised diagnostic algorithm

Tables 3 and 4 and Fig. 1 summarise the harmonised diagnostic approach. As the clinical presentation of CNS infections might be non-specific and overlap between different types of CNS infections and non-infectious complications, brain imaging is the first step to identify the clinical entity: focal infection, meningitis or encephalitis. Since the etiological spectrum differs significantly between these presentations, separate diagnostic algorithms are provided. Given the wide range of potential pathogens and the need for prolonged therapy in some cases (e.g., focal brain lesion), with possible frequent side effects or drug-drug interactions, pathogen identification is strongly recommended. Since diagnostic methods are constantly evolving, close collaboration with the microbiology and pathology laboratory is essential to ensure adequate samples are requested, acquired, and processed either internally or externally. Algorithms based on diagnostic stewardship should be provided to ensure optimal testing.

**Table 3:** Diagnostic work-up in patients with suspected brain focal lesion.

Steps	Diagnostic procedures	Actions and key considerations
1. Clinical suspicion	Physical examination identifying the presence of neurological complaints and, particularly, focal signs and symptoms	• Clinical presentation might be atypical or attenuated in immunocompromised patients • Evaluate GCS, assess a potential lesion’s position based on local deficits
2. Dedicated neuroimaging	• CT or preferably MRI with contrast and sequences T1, T2, FLAIR, DWI for identification of focal brain lesion(s)	• Exclude brain localisation of haematological malignancy or presence of other non-infectious aetiology (e.g., haemorrhage) • Evaluate the presence of oedema and the need for medical or neurosurgical treatment • Exclude increased intracranial pressure for CNS/CSF sampling feasibility • Evaluate concomitant presence of sinusitis or otitis: the presence of mastoiditis suggestive of bacterial origin; the presence of sinusitis suggestive of bacterial or fungal origin • Look for characteristic patterns of specific pathogens but consider that radiological presentation might be altered by the lack of inflammatory response
3. Initial work-up	**Medical history:** • Ongoing prophylaxis • Review of pre-HSCT serology for *T. gondii* • History of previous CNS and other infections • History of environmental and animal exposure and previous stays in endemic areas **Laboratory analyses:** • Complete blood count, inflammatory markers, routine parameters such as natrium, calcium, magnesium, TSH, Glucose • Blood level of azoles, if ongoing prophylaxis or therapy	Establish pre-test probability of different aetiologies based on: • Epidemiological risk factors (e.g., country of origin or travel history for tuberculosis, certain fungi or parasites) • *T. gondii* serology (CNS infection unlikely in patients with truly negative IgG) • Effective prophylaxis, such as mould-active azole and *Cryptococcus*-active antifungal prophylaxis, anti-toxoplasmosis prophylaxis (if TMP-SMX is used, the risk of nocardiosis is also lower) • Evaluate symptoms suggestive of systemic dissemination of infectious process localised elsewhere, e.g., lung infection, otitis, sinusitis, skin infection, etc • Evaluate the feasibility of CNS/CSF sampling: e.g., platelet count
4. Non-CNS microbiological tests	Establish pre-test probability of different aetiologies based on non-invasive microbiological results: • Blood cultures (relevant for e.g., *Nocardia* spp., *Fusarium* spp., bacterial abscess) • Blood *T. gondii* PCR and donor/recipient serology (if previous serology not available or negative) • Blood fungal antigens: GM, BDG • Blood PCR for fungi: *Aspergillus* spp., Mucorales, pan-fungal • Serum cryptococcal antigen • In case of lung lesions: BAL, if feasible	Exclude disseminated bacterial, fungal or parasitic infection
5. Lumbar puncture and CSF analysis	CSF analysis for cell count, protein, glucose, and tests for specific pathogens: • Direct microscopic examination (Gram, Ziehl-Nielsen, Calcofluor, Giemsa) • Culture for bacteria, mycobacteria, and fungi • Molecular assays for bacteria (syndromic panel or mono/oligoplexed PCRs) • *T. gondii* PCR • GM, BDG, cryptococcal antigen • PCR for *Aspergillus* spp., Mucorales, pan-fungal • PCR for *Mycobacterium tuberculosis* • EBV-PCR and JCV-PCR • NGS (16S and 18S/ITS sequencing and/or mNGS), if no diagnosis made with standard tests	• A lumbar puncture may provide important information for the microbiologic diagnosis of brain lesions in certain settings. • Prior to requesting and performing a lumber puncture, the potential diagnostic yield, associated risks (e.g., mass effect, intracranial pressure, etc.), and results of blood and other extra-CNS diagnostics should be considered to assess the potential benefit or not of such procedure • Normal CSF analysis does not exclude infectious focal brain infection, particularly if localised not in proximity of the ventricles, or due to prompt initiation of empirical or pre-emptive therapy • Cerebral PTLD and PML might present as brain lesions^52^, ^53^
6. Ancillary tests and evaluation of extra-CNS sites of infection	Chest and abdominal CT performed rapidly without delaying LP	• CT can lead to diagnosis of disseminated IFI or bacterial infection (e.g., nocardiosis, or more rarely, tuberculosis) • Echocardiogram should be performed if endocarditis is suspected
	PET Scan	To exclude relapse of haematological disease, if applicable and to document other sites of infectious involvement
	EEG	To detect seizures, intracranial hypertension
	Fundoscopy	To diagnose intracranial hypertension and detect the signs of endocarditis (if bacterial dissemination is suspected) or retinitis (e.g., in toxoplasmosis)
	ENT evaluation	To exclude otitis or sinusitis, perform targeted sampling for culture
	BAL in case of pulmonary lesions, with microscopy, cultures, and molecular tests for bacteria and fungi (e.g., PCR for *Aspergillus* spp., Mucorales, pan-fungal), GM	To confirm infection with pathogens that can disseminate to CNS, e.g., filamentous fungi, *Nocardia*, etc
	Immune reconstitution (CD4+/CD8+ T cell counts, IgG with subclasses, IgA, IgM)	To assess predisposition to infections
7. Invasive diagnosis of CNS infection	Brain lesion aspiration, biopsy or excision for histopathological exam and stain and culture for bacteria (including fastidious organisms and *Nocardia* spp.), fungi and mycobacteria; molecular assays (PCR, mNGS) for bacteria, fungi, and *T. gondii*	Send fresh samples to microbiology, since sensitivity is higher in fresh tissue than in paraffined specimens

16S, 16S ribosomal RNA gene; 18S, 18S ribosomal RNA gene; BAL, bronchoalveolar lavage; BDG, β-D-glucan; CD, cluster of differentiation; CNS, central nervous system; CSF, cerebrospinal fluid; CT, computed tomography; DWI, diffusion-weighted imaging; EBV, Epstein–Barr virus; EEG, electroencephalography; ENT, ear, nose and throat; FLAIR, fluid-attenuated inversion recovery; GCS, Glasgow Coma Scale; GM, galactomannan; HSCT, haematopoietic stem cell transplantation; IFI, invasive fungal infection; IgA, immunoglobulin A; IgG, immunoglobulin G; IgM, immunoglobulin M; ITS, internal transcribed spacer; JCV, JC virus; LP, lumbar puncture; mNGS, metagenomic next-generation sequencing; MRI, magnetic resonance imaging; NGS, next-generation sequencing; PCR, polymerase chain reaction; PET, positron emission tomography; PML, progressive multifocal leukoencephalopathy; PTLD, post-transplant lymphoproliferative disorder; T1, T1-weighted magnetic resonance imaging; T2, T2-weighted magnetic resonance imaging; TMP/SMX, trimethoprim–sulfamethoxazole; TSH, thyroid-stimulating hormone.

**Table 4:** Diagnostic work up in patients with suspected meningitis.

Step	Diagnostic procedure	Actions and key considerations
1. Clinical suspicion	Suspect meningitis in a patient with clinical picture of neurological impairment usually with fever, physical exam with GCS, identifying presence of neurological signs and symptoms, including meningeal signs	• Classic triad of fever, nuchal rigidity and mental status alteration might be absent • High clinical suspicion is important, as treatment must be provided urgently, preferably after CSF analysis if feasible
2. Initial work-up	**Medical history:** • Ongoing prophylaxis • Presence of post-HSCT complications • Environmental exposure **Laboratory analyses:** • Complete blood count, inflammatory markers, ammonia • Blood level of azoles if ongoing prophylaxis or therapy	Evaluate the following to establish the pre-test probability of different aetiologies: • Time after HSCT, presence of hypogammaglobulinemia ○ Late onset meningitis in patients with chronic GVHD or hypogammaglobulinemia can be due to encapsulated bacteria: *Streptococcus pneumoniae, Haemophilus influenzae, Neisseria meningitidis* • Environmental exposure (e.g., listeriosis outbreak, travel history to endemic areas, consumption of unpasteurised milk products, cold cuts and other foods potentially infected with *Listeria monocytogenes*) • Post-transplant prevention practices such as vaccination and prophylaxis • Previous history of CNS and other infections • Hyperammonaemia might be caused by bacteria such as *Ureaplasma* or *Mycoplasma*^54^, ^55^
3. Non-CNS microbiological tests	• 3 sets of blood cultures • Pneumococcal urinary antigen • Serum cryptococcal antigen	Exclude or confirm evidence of disseminated bacterial or fungal infection.
4. Neuroimaging	MRI, CT (if MRI not readily available)	• Exclude non-infectious aetiology • Exclude presence of meningoencephalitis compatible with viral aetiology • Exclude increased ICP, which is—contraindication for LP • Evaluate concomitant presence of sinusitis or otitis
5. Lumbar puncture and CSF analysis	CSF analysis for cells and differential, glucose, protein, and tests for specific pathogens: • Microscopic examination with stains for bacteria and cryptococcus • Culture for bacteria and fungi • PCR for bacteria and viruses • Pneumococcal antigen • Cryptococcal antigen • NGS (16S and 18S/ITS sequencing and/or mNGS), if no diagnosis made with standard tests	• Assess presence of classical patterns of bacterial meningitis (high PMN cell count, high protein, low glucose) versus viral pattern (lymphocytic predominance, normal protein and glucose), eosinophilic predominance may occur in meningitis due to *Coccidioides* spp. or a parasitic infection^43^, ^50^, ^56^, ^57^ • Diagnosis of viral meningitis is addressed in the companion paper
6. Ancillary tests	Fundus Consider radiological evaluation of extra-CNS sites of infection	Fundus: ICP assessment before LP

16S, 16S ribosomal RNA gene; 18S, 18S ribosomal RNA gene; CNS, central nervous system; CSF, cerebrospinal fluid; CT, computed tomography; GCS, Glasgow Coma Scale; GVHD, graft-versus-host disease; HSCT, haematopoietic stem cell transplantation; ICP, intracranial pressure; ITS, internal transcribed spacer; LP, lumbar puncture; mNGS, metagenomic next-generation sequencing; MRI, magnetic resonance imaging; NGS, next-generation sequencing; PCR, polymerase chain reaction; PMN, polymorphonuclear neutrophils; spp., species.

**Fig. 1:**
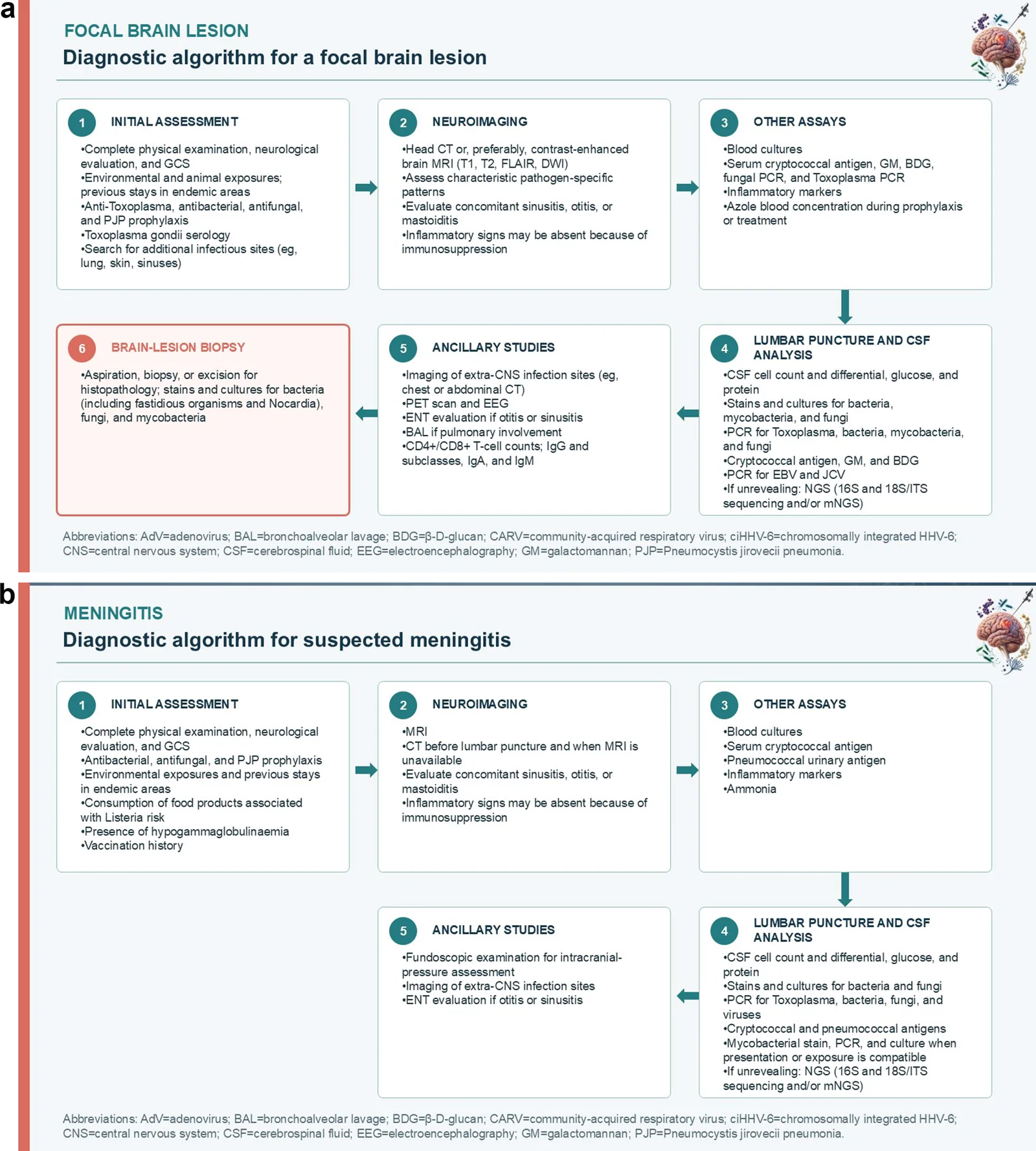
Harmonised diagnostic approach to (a) brain lesion or (b) meningitis in recipients of cellular therapy. Footnote: GCS, Glasgow Coma Scale; PJP, *Pneumocystis jirovecii* pneumonia; FLAIR, Fluid-Attenuated Inversion Recovery; DWI, Diffusion Weighted Imaging; GM, Galactomannan; BDG, Beta-D-Glucan; PCR, Polymerase Chain reaction; CSF, Cerebrospinal Fluid; EBV, Epstein–Barr Virus; JCV, JC Virus; NGS, Next Generation Sequencing; mNGS, Metagenomic NGS; PET, Positron Emission Tomography; EEG, Electroencephalogam; ENT, Ear Nose Throat; BAL, Bronchoalveolar Lavage; Ig, Immunogloboluins; ICP, Intracranial Pressure.

### Focal brain infection

The triad typical for brain abscess of headache, fever and focal neurological deficit or altered level of consciousness is present in approximately only 20% of immunocompetent patients on admission, but in immunocompromised hosts the clinical presentation might be even less specific.^1^, ^2^ In patients with brain abscess, neurologic signs and symptoms depend on abscess localisation, but can be mild and develop gradually, leading to late diagnosis, even at autopsy, in some cases.^1^, ^20^, ^23^ Suspicion of asymptomatic brain involvement should be raised when specific pathogens (e.g., *Nocardia* spp, fungi) are cultured outside the CNS, and brain imaging (preferably MRI) should be routinely performed in such cases.^58^

A proposed diagnostic investigation is outlined in Table 3. Upon clinical suspicion of focal brain infection radiological evaluation should be prioritised, preferably MRI.^3^, ^5^, ^6^, ^31^, ^56^ While a CT is easier to obtain and may exclude various causes such as bleeding or ischaemia, MRI with a protocol including T1- and T2-weighted images, diffusion-weighted imaging (DWI) and fluid-attenuated inversion recovery (FLAIR) is the diagnostic modality of choice for suspected CNS infections in HSCT recipients due to its superior sensitivity.^3^, ^5^, ^6^, ^31^, ^56^ Multiple lesions are frequent in cases of IFI (e.g., 35–86% of cases)^28^, ^29^, ^32^, ^33^, ^34^ or nocardiosis.^58^ The interpretation of radiological images should take into consideration that suppuration and other inflammatory responses might be absent or limited in neutropenic or severely immunocompromised patients even in case of focal bacterial lesions, and very limited in fungal or parasitic brain lesions.^59^ We recommend that a lumbar puncture should be performed, when safe and feasible, for CSF analysis and microbiological tests.^46^, ^60^, ^61^, ^62^, ^63^ There are still limited data in HSCT recipients on the performance of rapid molecular panels in bacterial infections,^64^ but NGS methods successfully identified mainly fungal pathogens in allogeneic HSCT recipients, while conventional diagnostic tests remained negative.^65^, ^66^ Cerebral post-transplant lymphoproliferative disorder (PTLD) and progressive multifocal leukoencephalopathy (PML) might present with brain lesions; hence, Epstein–Barr virus (EBV) and JC virus (JCV) PCR could be considered as adjunct tests in CSF.^52^, ^53^ The investigation of systemic infections should be performed both with imaging and additional microbiological diagnostic tests on blood samples, or BAL, if lungs are involved. In many cases, when a probable CNS infection is diagnosed at extra-CNS sites, further invasive procedures, such as a brain biopsy, can be withheld. Indeed, in cohorts of patients with CNS IFI, the etiological diagnosis of IFI was made on CSF or brain biopsies only in 8–33% of cases (Supplementary Table S4). If no microbiological diagnosis is obtained, prompt CT-guided aspiration or neurosurgical procedure, when deemed safe, is recommended. Craniotomy with resection of the abscess cavity may also be performed for diagnostic and therapeutic purposes. In contrast to the general population, with 10% of post-procedure complications,^67^ there are few data on the safety of stereotactic biopsy in HSCT recipients with neutropenia or thrombocytopenia, although it has been successfully used to confirm the diagnosis in patients with haematologic malignancies presenting with cerebral IFI (Supplementary Table S4). Empirical treatment may be withheld in clinically stable patients until after diagnostic procedures, which should be performed as early as possible.

### Meningitis

In immunocompromised hosts, fever and headache or mental status changes are the most frequent presenting signs and symptoms of infectious meningitis, not necessarily appearing altogether,^37^ hence vigilance and high clinical suspicion are required to make the diagnosis of meningitis accurately and promptly. Nuchal rigidity and impaired consciousness, for example, were less common in HSCT patients with neurolisteriosis than in other patient populations.^51^, ^68^

Initial assessment should start with obtaining blood cultures and performing brain imaging to exclude other aetiology and increased intracranial pressure, followed by a lumbar puncture, as detailed in Table 4. Although pleocytosis and increased protein (>5 white cells/μL in adults or above age-related norms in children) in the CSF are the mainstays of meningitis diagnosis, lower CSF cell counts and protein levels can be observed in immunocompromised patients compared to the general population due to decreased inflammatory response.^51^, ^68^ Polymorphonuclear predominance, high protein and low glucose levels in CSF were absent in 14% of HSCT patients with bacterial meningitis.^37^ Diagnostic microbiological tests on CSF should guide further management. In a patient with probable meningitis, blood culture positive for a typical community-acquired pathogen, like *S. pneumoniae,* establishes a microbiological diagnosis when pre-treatment CSF samples are unavailable. Similarly, cryptococcal antigen in blood is frequently positive in patients with cryptococcal meningitis. Multiple metanalyses in different patient populations, mainly with meningoencephalitis, reported that mNGS may allow for the diagnosis of CNS infections by detecting multiple, including rare, pathogens with a sensitivity in the range of 70%, with a specificity in the range of 93%.^69^, ^70^, ^71^ Therefore, mNGS may allow more microbiologically confirmed diagnoses in the future also in the HSCT setting, although its use remains still limited in clinical practice today.

## Empirical treatment and patient management

Due to the presence of the blood–brain barrier, antimicrobial treatment should include optimally dosed drugs (usually high dose when relevant) with CNS penetration, and drug interactions should be considered.^72^, ^73^, ^74^

### Focal brain infections

The recent ESCMID 2024 guidelines on brain abscess recommend empirical regimens for severely immunocompromised hosts with a combination of a third-generation cephalosporin plus metronidazole, trimethoprim–sulfamethoxazole, and voriconazole, with meropenem offered as an alternative antibacterial treatment if resistant Gram-negative bacteria are suspected.^47^ The German guidelines dedicated to patients with haemato-oncological diseases prioritised early pathogen-directed therapy, with upfront assessment of brain biopsy/neurosurgical resection feasibility, and empirical/pre-emptive therapy targeting bacteria with 3rd generation cephalosporin and ampicillin or alternatively meropenem with or without vancomycin, fungi with voriconazole or isavuconazole, and toxoplasma with pyrimethamine plus sulfadiazine.^75^

In patients with presentations which are not severe or life-threatening, the panel recommends that therapy should be chosen based on rapid results of diagnostic procedures. In the remaining cases of focal lesion(s), while awaiting etiological diagnosis, broad-spectrum empirical therapy should cover the most likely pathogens. The EBMT panel recommends high-dose TMP-SMX and imipenem-cilastatin or meropenem to cover *T. gondii* and *Nocardia* spp, while offering a broad antibacterial empirical coverage (Table 5). For empirical antifungal coverage, we recommend high-dose lipid formulation amphotericin B, particularly in a breakthrough infection case during broad-spectrum triazole prophylaxis, or isavuconazole or voriconazole in patients not receiving mould-active prophylaxis. Steroids should be used only in the presence of oedema, while immunosuppressive therapy reduction, if feasible, could be beneficial.

**Table 5:** Empirical therapy of focal brain infection and meningitis in recipients of cellular therapy.

Agents^a^	Comments
**Empirical therapy in case of focal brain lesion**	
High-dose TMP-SMX (15–20 mg of TMP/daily) and imipenem-cilastatin (500 mg q6 h) or meropenem (2 g every 8 h)	• High-dose TMP-SMX provides coverage in case of toxoplasmosis, and TMP-SMX and carbapenem provides coverage against common bacteria and *Nocardia* spp. • Most data on nocardiosis is based on imipenem and not meropenem treatment
L-AmB 5 mg/kg or isavuconazole or voriconazole if no azole-resistant fungus is suspected, and aspergillosis is strongly suspected	• The recommendation for L-AmB as first line empirical antifungal treatment is based on: ○ the increasing prevalence of non-*Aspergillus* spp., particularly in case of previous triazole use with high rates of breakthrough fungal infections reported in the recent years, ○ the increasing prevalence of azole resistance among *Aspergillus* spp. in different geographical regions, ○ literature data review revealing that AmB products are more frequently administered (69.3%, range 52%, 86%) compared to voriconazole (mean 53.5%, range 19%, 83%) either as monotherapy (mean of 54.4% of cases, range 18%, 86%) or in combination (51.9% of cases, range 17%, 82%); the most used antifungal combination therapy was liposomal amphotericin-B with voriconazole (45–63%) (Supplementary Table S3) • Isavuconazole or voriconazole should be used if no resistant fungus is suspected, and isavuconazole can be used in case of inability to administer L-AmB (e.g., severe renal dysfunction, allergy) • TDM of voriconazole should be performed to assure efficacy and limit toxicity (target range 2–5 mg/L); isavuconazole TDM might be used to assure efficacy • If a brain tissue biopsy can be obtained within 24–48 h and patient stable clinically, empirical antifungal therapy may be withheld
General concepts on antimicrobial stewardship principles	• Treatment should be adjusted based on pathogens identified from non-CNS sites highly likely to cause brain lesions or imaging presentation consistent with a type of pathogen(s) • As soon as a pathogen is identified, de-escalation of empirical antibacterial and/or antifungal treatment should be applied
**Empirical therapy in case of meningitis**	
Pre-engraftment meningitis or post-engraftment meningitis in repeatedly or chronically hospitalised patients: ampicillin (12 g/d) and vancomycin (15–20 mg/kg q8–12 h) together with or cefepime (2 g q8 h) OR, if resistant Gram-negative likely, meropenem (2 g q8 h)	• Pre-engraftment meningitis: ampicillin should be added to meropenem, if listeriosis cannot be rapidly excluded, considering possible failure of meropenem in treatment of listeriosis^76^, ^77^ • Steroid therapy is not recommended
Post-engraftment community-acquired meningitis: ceftriaxone (2 g q12 h) or cefotaxime (2 g q4–6 h) and ampicillin ± vancomycin	• Consider vancomycin based on the local rate of ceftriaxone resistance among *Streptococcus pneumoniae* • Adjunctive steroid therapy is not routinely recommended but can be considered in patients with late post-cellular therapy meningitis when *Streptococcus pneumoniae* or *Haemophilus influenzae* are likely. The timing of administration should be as close as possible, preferably before, the first antibiotic dose, and maximum up to 12 h after the first antibiotic administration
Ventriculoperitoneal shunt-associated meningitis: vancomycin + anti-pseudomonal beta-lactam, such as cefepime (2 g q8 h), ceftazidime (2 g q8 h), or meropenem (2 g q8 h)	The choice of beta-lactam will depend on patient’s risk of resistant *P. aeruginosa*

CNS, central nervous system; L-AmB, liposomal amphotericin B; TDM, therapeutic drug monitoring; TMP-SMX, trimethoprim-sulfamethoxazole.

a Detailed data on adult and paediatric dosage are in Supplementary material in Supplementary Tables S6 and S7 for normal renal function; due to the presence of blood brain barrier, antimicrobial treatment should include optimally dosed drugs (usually high dose when relevant) with CNS penetration, and drug interactions should be considered.^72^, ^73^, ^74^

### Meningitis

In immunocompromised patients, as addressed in ESCMID,^56^ WHO,^49^ UK,^50^ and German^75^ guidelines, empirical treatment with intravenous ceftriaxone or cefotaxime and ampicillin is recommended,^75^ aiming to cover community-acquired bacteria (*S. pneumoniae*, *Haemophilus influenzae* and *Neisseria meningitides*) as well as *L. monocytogenes*.^75^, ^78^ The German guidelines recommend meropenem with or without vancomycin as an alternative. The international guidelines recommend immediate antibiotic therapy administration if brain imaging or lumbar puncture is delayed.^43^, ^56^

The panel recommends that antibiotic therapy must not be delayed by neuroimaging or lumbar puncture. In patients with pre-engraftment meningitis, empirical treatment choices should follow febrile neutropenia treatment guidelines, considering pathogens from previous bacteraemia and drugs with CNS penetration (Table 5). We recommend the same regimen for repeatedly or chronically hospitalised patients with post-engraftment meningitis. If Gram-negative bacteria resistant to third and fourth generation cephalosporins/ESBL-producing are likely based on previous colonisation or infection, meropenem should be prioritised. In patients with contraindications to vancomycin, such as a severe hypersensitivity reaction, linezolid can be considered instead. In patients with community-acquired post-engraftment meningitis, we recommend treatment with ceftriaxone/cefotaxime with ampicillin for empirical therapy. Vancomycin should be added as per local meningitis guidelines based on *S. pneumoniae* resistance rates. Anti-listeriosis coverage for patients with penicillin allergy should include TMP-SMX; meropenem is another alternative. Other indications for empirical meropenem include allergy to cephalosporins, or meningitis developing in the context of Gram-negative bacteraemia resistant to narrower-spectrum beta-lactams. Patients with ventriculoperitoneal shunt-associated meningitis should be treated as recommended by international guidelines, with vancomycin together with an anti-pseudomonal beta-lactam based on the susceptibility of the potential Gram-negative pathogen, as described above. For patients in whom viral meningoencephalitis cannot be excluded, empirical treatment with high-dose intravenous acyclovir is recommended, aiming to cover HSV (see dedicated companion paper for details). Considering the rarity of cryptococcal meningitis and wide availability of fast and reliable diagnostic tests, routine empirical antifungal coverage against cryptococci is not recommended in patients with meningitis.

### Adjunctive steroid treatment in bacterial meningitis

There is no data regarding the benefits and risks of adjunctive steroids in patients following HSCT. German guidelines addressing patients with haemato-oncological diseases do not recommend adjunctive steroids in general but recommend using them in patients with *S. pneumoniae* or *H. influenzae* meningitis, although they do not address the timing of steroid treatment in this situation.^75^ The unique aspects of meningitis in HSCT recipients should be considered, as discussed in Table 5. Few reported cases of early pre-engraftment post-HSCT meningitis were caused by bacteria for which the benefit of steroids has never been reported and probably could not be expected given profound immunosuppression typical for this period. Post-engraftment meningitis can occur in the context of GVHD in severely immunosuppressed patients, some already receiving steroids. Moreover, due to the blunted immune response in these patients, inflammation-mediated damage is unlikely. Data on adjunctive steroids in patients with *L. monocytogenes,* a typical pathogen causing meningitis in immunocompromised patients, are controversial. Improved outcomes have been reported in patients receiving adjunctive dexamethasone in a prospective multicentre study from the Netherlands (almost half were immunocompromised patients)^79^ and Sweden (specifically in immunocompromised patients).^80^ On the contrary, increased mortality was associated with steroid use in patients with neurolisteriosis in a national prospective French study.^68^ Two other prospective multicentre studies from Spain and the Netherlands,^81^, ^82^ one retrospective single centre Spanish study,^83^ and meta-analysis of six studies^84^ demonstrated no association between steroids and outcome. Finally, potential benefits of adjunctive steroids can be theoretically expected in patients not treated with steroids for other indications, with suspicion of late post-transplant meningitis due to *S. pneumoniae* or *H. influenzae.* As CSF analysis can be unavailable at the time of antibiotic administration (due to the need for immediate empirical therapy and avoiding delay while awaiting the imaging results), the initial decision on adjunctive steroids can be made first based on the risk factors assessment, such as partial immune reconstitution, hypogammaglobulinemia, especially with incomplete vaccination history. Steroids should still be considered as soon as possible, and within 12 h since the first antibiotic dose, if CSF analysis is compatible with *S. pneumoniae* or *H. influenzae type B* aetiology (based on high CSF WBC count, typical Gram stain, positive pneumococcal antigen in CSF or positive rapid panel diagnosis).^85^

The EBMT panel does not recommend routine administration of steroids as a part of empirical therapy in cellular therapy recipients with suspected bacterial meningitis, but recommends considering adjunctive steroids when *S. pneumoniae* and *H. influenzae* are likely based on risk factors (such as hypogammaglobulinemia, unvaccinated patients, asplenia, chronic GvHD not on steroid treatment) or laboratory data compatible with typical findings (high CSF WBC count, typical Gram stain, positive pneumococcal antigen in CSF).

## Targeted therapy and secondary prophylaxis

Targeted therapy for each cause of focal infection or meningitis is beyond the scope of this paper and should follow specific guidelines, preferably for the immunocompromised population.^48^, ^86^, ^87^, ^88^ Targeted therapies for selected main pathogens relevant in HSCT recipients are provided in Supplementary Table S6.^72^, ^73^, ^74^ The treatment length will depend on the final microbiological diagnosis, the clinical and radiological response, and the patient’s immunological reconstitution. An intraventricular or intrathecal antibiotic administration can be considered in refractory and critically ill patients poorly responding to systemic therapy with meningitis due to multidrug-resistant bacteria, especially those with VPS-associated infections, although the large-scale data are lacking and there are inherent procedural risks.^89^ Secondary prophylaxis should be provided when immunodeficiency persists in patients with resolved IFI or *T. gondii* infection, following guidelines for specific pathogens, until the immunosuppression has resolved.^48^, ^86^, ^87^, ^88^

## Harmonised response assessment and responses definition

The recommended assessment and response definitions were developed aiming to provide a standard reporting of response in clinical studies. Response definitions are provided in Table 6. The presence or absence of neurological sequelae should be reported separately, as complete or partial response with or without sequelae, since there is no association between the length of therapy and the probability of experiencing improvement in neurological sequelae.

**Table 6:** Summary of response definitions at harmonised endpoints post treatment initiation (2 and 4 weeks for non-tuberculous meningitis and 6 weeks for focal brain infections; with additional evaluation at 12 weeks for brain lesions due to opportunistic pathogens, such as *Nocardia* spp., *Toxoplasma gondii*, and fungi).

Overall assessment	Response category^a^	Definition for focal lesion—6 and 12 weeks	Definition for meningitis—2 and 4 weeks
Success	Complete response, with or without neurological sequelae^b^	Patient alive, complete resolution of clinical signs and symptoms and radiological findings (scarring might be present)	Patient alive, no evidence of active infection based on clinical, laboratory (blood inflammation markers; CSF analysis if available) and imaging parameters
	Partial response, with or without neurological sequelae^b^	Patient alive, partial resolution of clinical signs and symptoms and of radiological findings^c^	Cases with clinical improvement or stable residual neurological deficits, with suspicion of not fully controlled infection based on clinical signs (e.g., persistent fever, headache), blood inflammatory markers (CRP), and/or radiological findings^c^^,^^d^
Failure	Stable disease	No change in clinical signs and symptoms and/or radiological findings^e^	No change in clinical signs and symptoms and/or radiological findings^e^
	Progression	Worsening of clinical symptoms or signs of disease and/or imaging findings	Worsening of clinical symptoms or signs of disease and/or imaging findings
	Death	To be reported as due to CNS infection or as death not related to CNS infection (in that case indicate if CNS infection was in complete or partial response or stable at the time of death)	To be reported as due to CNS infection or as death not related to CNS infection (in that case indicate if CNS infection was in complete or partial response or stable at the time of death)

CNS, Central Nervous System; CSF, Cerebrospinal Fluid; CRP, C-Reactive Protein.

a The global assessment of response should take into consideration pathogen-specific clinical, microbiological, and radiological domains. Microbiological re-evaluation is rarely performed in case of focal lesions. Separating clinical, microbiological and radiological response might be challenging and discrepancies might exist. In such cases, appropriately evaluated (usually with culture) microbiologically documented failure is the most relevant, since radiological response might worsen in case of immune reconstitution, and clinical presentation might be influenced by CNS symptoms of other aetiology.

b The presence or absence of neurological sequalae should be reported separately from the overall response assessment since there are no associations between the length of therapy and the probability to experience improvement of neurological sequelae.

c Such patients are suspected to have complications, such as abscess, sinus vein thrombosis or subdural effusion/empyema that require assessment by imaging, prolonged treatment course and sometimes neurosurgery interventions.

d Partial response might also reflect slow immune reconstitution.

e While stable disease is considered as failure when evaluating response in clinical trials, from the clinical point of view a stable response might reflect slow immune reconstitution and, in some cases, might not require therapeutic changes if other causes of the lack of response were excluded (e.g., insufficient drug levels, etc).

### Focal brain infection

The timing of assessment depends on the brain lesion aetiology, clinical response, particularly in cases of empirical treatment when the pathogen remains unknown, and the overall patient immune status. For instance, in patients with clinically improving nocardiosis or toxoplasmosis, imaging should be repeated after approximately 4–6 weeks of therapy. Imaging should be repeated earlier, usually at 2 weeks or as clinically indicated, in patients not improving and/or without definitive diagnosis (e.g., presumptive toxoplasmosis based on imaging and risk factors), to guide further diagnostic procedures or therapeutic changes.^88^, ^90^, ^91^ We propose a harmonised timing of formal overall response assessment to be performed at 6 weeks for all types of pathogens, using criteria applied to evaluate IFI. Additional assessment should be performed at 12 weeks after diagnosis of focal brain lesions due to opportunistic pathogens, such as *Nocardia* spp, *T. gondii*, and fungi.

### Meningitis

Response is assessed mainly based on clinical and laboratory improvement while receiving therapy with appropriately dosed antimicrobials penetrating the CNS and active against the identified pathogen in cases of microbiologically documented infections, or against a presumed pathogen without microbiological documentation. In not improving patients, especially without microbiological documentation, additional diagnostic efforts should be undertaken, such as imaging (brain MRI, total body scan or PET-scan to exclude other localisations), advanced microbiological tests, search for an alternative explanation of non-infectious origin; and the antimicrobial coverage broadening should be considered. Repeated CSF analysis is not routinely recommended to confirm pathogen eradication, except in specific situations similar to the general population including: lack of clinical response after 48 h of appropriate antimicrobial therapy; *S. pneumoniae* meningitis non-susceptible or with borderline susceptibility to beta-lactams; Gram-negative bacilli meningitis; meningitis in the presence of ventriculoperitoneal shunt; and cryptococcal meningitis if there is persistent CSF pressure elevation ≥25 cm and symptoms until the CSF pressure and symptoms have been stabilised for >2 days.^43^, ^89^, ^92^ As the standard treatment course for bacterial non-tuberculous meningitis is usually 10–21 days, we recommend response assessment at 2 and 4 weeks after treatment onset.

## Conclusions

This paper provides definitions, epidemiology and diagnostic approaches to both focal brain infections and meningitis in HSCT recipients, which are summarised in Supplementary Table S8. Current knowledge gaps include a lack of updated epidemiology data on focal CNS infections and meningitis in the era of mould-active prophylaxis and the frequent use of fungal biomarkers and agnostic molecular methods, such as metagenomics. Successful uptake depends on addressing known facilitators (adequate time, training, and organisational leadership) and barriers (limited resources hampering sustainable implementation, insufficient training, and competing priorities) at the individual, organisational, and system levels.

Further studies are warranted to evaluate the epidemiology using standardised definitions and the performance of novel diagnostic methods, including how their timely prompt results could contribute to shifting from broad- spectrum empirical therapy to early optimal targeted treatment for CNS infections.

## Contributors

All co-authors conceptualised the subject for this workshop; participated in the face-to-face meeting and wrote the original manuscript draft; played an important role in interpreting results, revised the manuscript, approved the final version, and agreed to be accountable for all aspects of the work in ensuring that questions related to the accuracy or integrity of any part of the work are appropriately investigated and resolved. MM, DA, DN, and FB performed the literature searches, while all panel members participated in evidence appraisal and final expert consensus.

## Declaration of interests

MM: Speaker and advisory board member for Gilead, Mundipharma, Pfizer, and Shionogi, not related to this work.

DA: Speaker for MSD, Shionogi and Takeda, not related to this work.

DNe: Grants from Mundipharma and Takeda, consulting fees from Shionogi and MSD, honoraria for lectures from Gilead, MSD and Takeda, all payments made to his institution, not related to this work.

FB: Advisory board AMGEN.

JLP: Advisory support: MSD; AstraZeneca; GSK; Pfizer; Moderna. Support for meeting attendance: MSD, Pierre Fabre.

MSH: No conflicts of interest related to this work.

RC: No conflicts of interest related to this work.

SC: No conflicts of interest related to this work.

DNa: Grants from BioMérieux, Pfizer, and Vircell SL, consulting fees from BioMérieux, and honoraria and meeting support by BioMérieux, Abbott, Pfizer and MSD.

FO: No conflicts of interest related to this work.

AR: No conflicts of interest related to this work.

PL: Honoraria for lectures and consulting fees from MSD, Takeda, and Symbio Pharmaceuticals.

ISO: No conflicts of interest related to this work.

IYA: No conflicts of interest related to this work.

JS: No conflicts of interest related to this work.
