# Supplementary material for: Syndrome-oriented CNS infectious complications after haematopoietic stem-cell transplantation and CAR T-cell therapy, part 2—focal brain infections and meningitis of bacterial, fungal, and parasitic origin: recommendations from the EBMT Practice and Harmonization Committee
Source: eClinicalMedicine. 2026 Nov 5;101:104311. doi: 10.1016/j.eclinm.2026.104311 (PMC13760377; doi:10.1016/j.eclinm.2026.104311)
Supplement: Supplementary file 1 [file mmc1.docx]

**Title page**

Syndrome-oriented CNS infectious complications after Hematopoietic Stem Cell Transplantation and CAR‑T Therapy: Part 2. Approach to focal brain infections and meningitis of bacterial, fungal and parasitic origin. Recommendations from the EBMT Practice & Harmonization Committee.

**Supplement Material**

**Contents**

[**Literature search**	3](#_Toc243376092)

[**Detailed results of literature review**	3](#_Toc243376093)

[**Pathogen-specific data**	3](#_Toc243376094)

[**Focal brain lesions**	4](#_Toc243376095)

[*Nocardia* spp.	4](#_Toc243376096)

[Toxoplasmosis	5](#_Toc243376097)

[Other parasites	5](#_Toc243376098)

[Fungi	6](#_Toc243376099)

[**Meningitis**	7](#_Toc243376100)

[*Listeria monocytogenes*	7](#_Toc243376101)

[*Mycobacterium tuberculosis* complex	7](#_Toc243376102)

[Cryptococcal meningitis	8](#_Toc243376103)

[Other	8](#_Toc243376104)

[**Tables**	9](#_Toc243376105)

[Table S1.	9](#_Toc243376106)

[Table S2.	15](#_Toc243376107)

[Table S3.	18](#_Toc243376108)

[Table S4.	21](#_Toc243376109)

[Table S5.	23](#_Toc243376110)

[Table S6.	25](#_Toc243376111)

[Table S7.	27](#_Toc243376112)

[Table S8.	28](#_Toc243376113)

[**References**	29](#_Toc243376114)

# **Literature search**

A comprehensive literature search was conducted in PubMed to identify relevant studies published between January 1, 2000 and September 2025. The search strategy combined Medical Subject Headings (MeSH) terms and free-text keywords related to central nervous system (CNS) infections and hematopoietic transplantation. Search terms included “brain abscess,” “brain infection,” “cerebral lesion,” “CNS infection,” “central nervous system infection,” “meningitis,” and “meningoencephalitis,” as well as specific entities such as “fungal meningitis,” “fungal meningoencephalitis,” “fungal central nervous system,” “toxoplasma,” “toxoplasmosis,” “ameba,” “amoeba,” “parasitic infection,” and “neurocysticercosis.” These terms were combined using Boolean operators with transplantation-related MeSH terms, including “Hematopoietic Stem Cell Transplantation,” “Bone Marrow Transplantation,” “Transplantation, Autologous,” and “Transplantation, Allogeneic,” as well as free-text terms such as “stem cell transplant*,” “bone marrow transplant*,” “hematopoietic stem cell transplant*,” “HSCT,” “BMT,” “CAR-T*,” and “transplant recipients.” Additional targeted searches incorporated pathogen-specific MeSH terms (e.g., “Streptococc*”) in combination with CNS-related terms (“brain,” “cerebr*,” “meningitis”). Searches were limited to studies conducted in humans and articles published in English. For fungal and parasitic infections, case reports and small case series including fewer than five patients were excluded. The reference lists of relevant articles were manually reviewed to identify additional eligible studies.

# **Detailed results of literature review**

# **Pathogen-specific data**

Detailed data of identified studies that report epidemiology (rate, etiology) and outcome of parenchymal lesions and meningitis in the studies on CNS complications after Hematopoietic Stem Cell Transplantation (HSCT), including the rate of CNS involvement in studies on specific pathogens after HSCT in reported in table S1.

It includes also an autopsy-based study from Brazil, which reported CNS data from 180 autopsies (177 allo-HSCT, 3 auto-HSCT) performed in a cohort of 845 allo-HSCT and auto-HSCT recipients, of who 317 died and 196 had autopsy performed (in 180 CNS data were available). Overall, 27CNS infections were identified, 3 bacterial (incidence 0.36% in the whole HSCT cohort), 16 fungal (incidence 1.9%) and 8 due to Toxoplasma (incidence 0.95%). ^1^

# **Focal brain lesions**

## *Nocardia* spp.

Although nocardiosis remains relatively rare, reported in 0.3-1.7% following allo-HSCT and 0.2% following auto-HSCT ^2-4^, CNS involvement is a frequent manifestation, with *Nocardia* spp. representing one of the most common bacterial causes of focal parenchymal infections in immunocompromised hosts, including after HSCT. In a multinational EBMT study, nocardiosis occurred at a median of 8 (IQR: 4–18) months following HSCT ^5^. CNS involvement was the second most common presentation following the lungs and was reported in 37% of cases. Two-thirds of patients presented with multiple brain abscesses, and one-third with a single brain lesion. Brain involvement typically (90%) occurred in the setting of disseminated infection ^5^. Of note, a third of patients with CNS nocardiosis lacked neurological symptoms, thus brain imaging, preferably MRI, should be routinely performed in HSCT recipients with nocardiosis ^6^. Risk factors for nocardiosis have included male sex, lymphocyte counts < 500/µL, impaired T-cell recovery, administration of tacrolimus, recent CMV infection and not using trimethoprim-sulfamethoxazole (TMP-SMX) prophylaxis ^7-10^. *Nocardia farcinica*, frequently resistant to ceftriaxone and in some cases also to meropenem, was the most frequent species reported in CNS nocardiosis ^11^, justifying the use of imipenem-cilastatin (preferred) or high-dose meropenem in combination with TMP-SMX until susceptibility results are available. Relapse of CNS nocardiosis has been reported in 3-12.5% of patients ^5,12^. One-year mortality in HSCT patients with nocardiosis was approximately 40%, brain involvement has not been associated with worse prognosis ^5^.

## Toxoplasmosis

The epidemiology, definition, diagnosis and management of toxoplasmosis after HSCT have been recently reviewed ^13^. Among studies on CNS infections in HSCT recipients, neurotoxoplasmosis rate ranged from 0 to 3.1%, accounting for 0-74% of all CNS infections (Table 2 and S5). The most relevant risk factor is the recipient’s pre-transplant positive serology. Anti-*Toxoplasma* TMP-SMX prophylaxis is the major intervention to reduce risk of disease ^14^, even though cases of breakthrough toxoplasmosis in patients on TMP-SMX prophylaxis have been reported ^15,16^. Higher risk of CNS toxoplasmosis was associated with atovaquone, dapsone, and pentamidine prophylaxis compared to TMP-SMX ^17,18^. CNS involvement is the most frequent clinical manifestation of *T. gondii* reactivation leading to death in 20-100% of cases, in both adult ^14,16,19,20^ and paediatric cohorts ^21,22^. Median time to onset of CNS toxoplasmosis was 65 days (range, 13-280) after HSCT ^14,16,17,19,23,24^. Brain CT scan typically reveals multiple bilateral lesions located at the corticomedullary junction and basal ganglia, usually hypodense with ring enhancement and surrounding vasogenic oedema after contrast administration, although cases with normal or atypical initial CT have been reported ^20^. MRI is more sensitive than CT and thus preferred for early diagnosis. It typically shows hyperintense lesions on T2-weighted sequences with non-specific space-occupying characteristics, often with ring enhancement after contrast ^14,16,25,26^. CSF findings may show mild mononuclear pleocytosis, increased protein concentration, and normal glucose levels, and detection of *T. gondii* DNA by PCR in CSF represents the cornerstone of microbiological diagnosis ^16,20^. Recent ECIL-9 guidelines recommend universal testing for CNS involvement with preferably MRI in patients with positive *T. gondii* blood PCR, as parasitaemia often precedes clinical disease and early CNS lesions may be asymptomatic ^13^.

## Other parasites

*Acanthamoeba*, a free-living amoeba, may cause granulomatous amoebic encephalitis (GAE) in rare cases, mainly in immunocompromised hosts. In the transplant setting, it occurred within the first year after HSCT, most frequently in the context of severe T-cell immunosuppression, GvHD, and high-dose steroid therapy (Table S6)^27-31^. The mortality rate in transplant recipients was above 90%, with very short interval between CNS symptom onset and death (median 8 days, range 7-42 days). Diagnosis was based on brain biopsy or autopsy. Treatment included various not-standardized multidrug combination therapy, with almost universal progression despite therapy ^29,30,32^.

CNS infections caused by *Taenia solium,* i.e. neurocysticercosis, have been reported as very late post-HSCT complication ^33-35^. Favourable outcomes with standard anti-parasitic drugs plus steroids treatment were reported ^36^.

## Fungi

Fungi are the main cause of focal brain infections reported in allo-HSCT recipients. Of note, in an autopsy study of 180 of 371 deceased allogeneic (allo-HSCT) or autologous HSCT (auto-HSCT) recipients, most of whom had severe aplastic anaemia or chronic myelogenous leukaemia and died within 3 months after HSCT, the focal brain infection rate was particularly high (13%), due to predominantly fungal pathogens,^1^ while other studies reported the *T. gondii* infection predominance.^20,37,38^ Prior IFI, especially pulmonary aspergillosis, was the strongest risk factor for CNS IFI after allo-HSCT.^39^ Recent studies using NGS diagnostics in HSCT setting, identified mainly fugal pathogens. In a retrospective study of 20 all-HSCT recipients from China, 8 fungal pathogens, including 2 cases of *Fusarium* spp., one case of Mucorales, and one of *Aspergillus niger*, were identified only by mNGS, while conventional diagnostic tests remained negative ^40^. Similarly, in another prospective study from China including 53 allo-HSCT recipients, mNGS identified *Aspergillus* spp., *Streptococcus pneumoniae*, and *Rickettsia* spp., while conventional diagnostic remained negative ^41^. Overall mortality was up to 100% in the studies published prior to 2017, while among studies published after 2017, 30 to 84-day mortality was in the range of 34.5% to 51.8%, with overall mortality up to 92% (Table S3). This may, in part, be attributed to earlier diagnosis and prompt initiation of effective treatment.

# **Meningitis**

## *Listeria monocytogenes*

Listeriosis is one of the most common causes of bacterial meningitis in cancer and HSCT patients ^42-44^. The incidence of listeriosis has ranged from 0.35 to 0.58% following HSCT, more frequent after allo-HSCT vs. auto-HSCT ^45-48^. CNS involvement was reported in one third of cases ^49,50^. In an international EBMT study, the median onset of CNS listeriosis was 13 (IQR 2-19) months post-HSCT ^50^. Abnormal CNS imaging was reported in roughly half of cases and included meningitis, meningoencephalitis and focal lesions. Cerebrospinal fluid (CSF) analysis typically shows polymorphonuclear (PMN) predominance (79% of cases), low glucose (38%), and may show normal protein levels. Gram stain was positive in 43% of patients. The majority of CNS listeriosis cases presented as meningitis or meningoencephalitis, with brain abscesses due to this agent rarely reported ^44^. Patients with CNS infection due to *Listeria monocytogenes* may present with rhombencephalitis, presenting with ataxia, seizures, or cranial nerve palsy ^51^. Primary risk factors for listeriosis after HSCT included undergoing ≥2 HSCT and having lymphopenia, while there was no difference in risk based on previous exposure to penicillins, TMP-SMX, or fluoroquinolones. Mortality is notably higher in patients with CNS involvement ^49^, with a 27% three-month mortality rate compared to 13% in non-CNS listeriosis cases ^50^. For prevention of listeriosis, patients should be counselled to avoid high-risk foods, including soft cheeses, unpasteurized dairy, undercooked processed meats, hot dogs, cold cuts, and refrigerated pate or smoked fish.

## *Mycobacterium tuberculosis* complex

Among all cases of tuberculosis after HSCT, already rare in most settings, CNS involvement was reported in 0-4% of cases. No cases of CNS involvement were reported in 7 studies including a total of 139 patients ^52-58^. In a review of 25 studies on tuberculosis infections in HSCT recipients reporting data on 122 patients, CNS involvement was present only in 1 patient ^59^. In an EBMT study reporting 42 cases of tuberculosis in a multicentre cohort, CNS involvement was reported in 2 (4%) cases ^60^. Finally, among 5 patients with active tuberculosis post CD19/CD22 target CAR T-cell therapy alone or following auto-HSCT in China, two were diagnosed with CNS involvement documented by PCR in CSF ^61^. In all patients with CNS tuberculosis, tuberculous meningitis is the most common clinical presentation, followed by tuberculous focal lesions, single or multiple, such as tuberculoma or tuberculous brain abscess ^62^.

## Cryptococcal meningitis

Cryptococcal meningitis is very rate in cellular therapy recipients. In a multicentre US study, among 875 HSCT recipients with invasive fungal infections, only 6 cases of cryptococcosis were identified (0.6%) ^63^. While in another multicentre study from the US on cryptococcosis in patients without HIV, of whom 49% had CNS cryptococcosis, only 1.3% (n=2) were HSCT recipients ^64^.

## Other

Although few reports are available in HSCT recipients, immunocompromised patients, particularly with B-depletions, might be at higher risk of meningoencephalitis due to *Borrelia, Legionella* or *Anaplasma.*^65^

**Tables**

## **Table S1.**

The epidemiology (rate, etiology) and outcome of parenchymal lesions and meningitis in the studies on CNS complications after HSCT, including the rate of CNS involvement in studies on specific pathogens after HSCT.

| Reference: first author, year (country) | Study design, population | Study population | Study main endpoint | Number and rate of any CNS infection | Number and rate of brain abscess; detailed etiology | Number and rate of bacterial meningitis; detailed etiology | Risk factors for CNS infection | Comments | Outcome |
| --- | --- | --- | --- | --- | --- | --- | --- | --- | --- |
| Prospective |  |  |  |  |  |  |  |  |  |
| Schmidt-Hieber 2026 (European multicentre) ^66^ | Prospective multicentre EBMT study | 2910 HSCT recipients (1735 allo-HSCT, 1175 auto-HSCT) | Any CNS complication after HSCT | 21, all after allo-HSCT (1.2%) | 8 toxoplasmosis (0.27% for any HSCT, 0.46% for allo-HSCT); 5 toxoplasmosis and 3 aspergillosis | 0.07% for any HSCT, 0.11% for allo-HSCT; *E. faecium* and *S. warneri* meningitis | Not reported specifically for CNS infections |  | 24- OS was higher for controls (82%, (95% CI 76–89%) vs 32% (95% CI 10–54%) for proven/  probable infectious CNSD patients |
| Schmidt-Hieber, 2020 (European, multicentre) ^67^ | Prospective multicentre EBMT study | 4,063 HSCT recipients (auto-HSCT and allo-HSCT); CNS disorders identified in 163 episodes | Spectrum of infectious vs non-infectious CNS disorders | CNS infection incidence: 2.6% allo-HSCT, 0.1% auto-HSCT | ND; Abscesses not separately quantified; fungal abscesses predominated (mainly *Aspergillus spp.*; also *Cryptococcus spp.*) | ND; bacterial CNS infections 9% of infectious cases; organisms not individually detailed | For-infectious vs. non-infectious disorders: steroid-based immunosuppression; recipient *Toxoplasma* seropositivity, EBV donor and recipients seropositivity | Prospective design; abscess phenotype included under fungal CNS infection | 30-day mortality highest for fungal CNS infections (87%, 20/23), and 50% (82/163) in all episodes |
| Retrospective |  |  |  |  |  |  |  |  |  |
| Mannina 2020 (Germany) ^68^ | Single-centre retrospective | 888 allo-HSCT recipients | Incidence of diverse  categories of CNS diseases | 9 (1%) | 1 toxoplasmosis | 0 | Not reported specifically for CNS infections | - | NRM higher in patients with CNS infection |
| Dowling, 2018 (USA) ^69^ | Single-centre retrospective cohort | 263 adult allo-HSCT recipients followed up to 5 years | Incidence, risk factors, and impact of NC after allo-HSCT | 1.1% (3/263 CNS infections) | 1 toxoplasmosis | 1 (0.4%) bacterial meningitis case (organism not specified); additional CNS infections: 1 viral encephalitis, | Not reported specifically for CNS infections | Modern-era cohort with low CNS infection incidence; infections were rare compared with haemorrhagic/metabolic events | CNS complications strongly associated with worse OS, PFS, and non-relapse mortality |
| Hanajiri, 2017 (Japan) ^70^ | Single-centre retrospective | 353 allo-HSCT recipients | Incidence and outcome of CNS infection after allo-HSCT | 5.5% at 5 years (n=17) | 0.5%: 2 cases of brain abscess: both due to *Staphylococcus capitis* | 5 (1.4%) meningitis cases: *Enterococcus spp.* (2), *Streptococcus pneumoniae* (1), *Staphylococcus haemolyticus* (1), VZV (1), plus 6 HHV-6 meningo-encephalitis cases | High-risk disease status | One of few studies explicitly reporting abscess pathogens | 3-year OS 33% with CNS infection vs. 53% in those without CNS infection (p=0.04) |
| Balaguer-Roselló, 2017 (Spain) ^71^ | Single-centre retrospective | 709 allo-HSCT (343 UCBT, 366 sibling donor) | Incidence and characteristics of CNS infections | 4.9% at 5 years (higher after UCBT) | 1.9% (14/709), 12 fungal: 8 *Aspergillus spp.,* 2 *Cryptococcus neoformans,* 1 *Scedosporium prolificans,* 1 Mucor; 2 bacterial: 1 *Nocardia spp., 1 Mycobacterium tuberculosis* (with hydrocephalus) | 2 (0.3%) cases of bacterial meningitis: 1 *Streptococcus pneumoniae*, 1 *Staphylococcus spp.* | UCBT; advanced disease; steroid use | Clear breakdown of fungal vs bacterial agents | 59% mortality attributable to CNS infection |
| Colombo, 2017 (Italy) ^72^ | Single-centre cohort (retrospective and prospective) | 452 adult allo-HSCT recipients | Incidence and risk factors of CNS NC | 9/452 (2%), any NC: 30 (6.6%) | 1% (5/452): 2 Toxoplasma gondii,  2 *Aspergillus*, 1 unspecified fungus | 2 cases of bacterial meningoencephalitis (0.4%): *Streptococcus* spp and *Pseudomonas* spp; 2 additional viral | Not reported specifically for CNS infections | In 23% of patients the etiology of CNS complication remained undefined | In 18/30 patients, NC was the major cause of death, among 7 patients with bacterial, fungal or parasitic CNS infection, only 1 patient with Aspergillus infection was alive with 30 days |
| Barba, 2009 (Spain) ^73^ | Single centre retrospective | 191 adult recipients of allo-HSCT after reduced-intensity conditioning | Incidence, characteristics, and etiologies of NC | 6 (3.1%): 4 viral, 1 fungal, in 1 infectious agent not reported; 23/191 (12%) any NC | ND, possibly 0.5%: 1 aspergillosis | 0/191 (0%) bacterial meningitis; 2 HSV, 1 adenovirus, and 1 suspected JCV | Not reported for CNS infection | All 5 infections reported as meningoencephalitis | 2 patients with meningoencephalitis died within 30 days |
| Teive, 2008 (Brazil) ^74^ | Single-centre retrospective | 1000 HSCT recipients; CNS infection rates calculated from per cent values | NC after HSCT | 2.5% (plus additional 2.5% of patients with herpes zoster radiculopathy); 11.3% among patients with CNS complications | Among those with CNS complications: 1% brain abscess; bacterial and fungal, no organism-level detail; 1% toxoplasmosis, 0.1% neurocysticercosis | Among those with CNS complications: 3% bacterial meningitis | Not reported | Both CNS and peripheral nervous system complications considered | All CNS complications caused 19% of deaths |
| Bjorklund, 2007 (Sweden) ^75^ | Case–control (late fatal infections) | 688 allo-HSCT survivors >6 months | Risk factors for fatal late infections | CNS infection caused 11% of 44 patients with any fatal late infections | Abscess etiology not specified | Bacterial, viral and fungal CNS infections pooled | Acute and chronic GVHD; CMV; unrelated donor; TBI | Study focused on fatal infection; no microbiological results | Not applicable |
| Denier, 2006 (France) ^20^ | Single-centre retrospective cohort | 361 HSCT recipients (116 allogeneic, 245 autologous) | Spectrum and prognosis of NC after HSCT | 4.2% (15/361); 10.3% (12/116) after allo-HSCT and 1.2% (3/245) after auto-HSCT | 2.4% (9/361): 5 Toxoplasma, 2 fungal (1 Trichoderma), 2 of undetermined etiology | 2 (0.6%): 1 pneumococcal meningitis (late, post-splenectomy); 1 of undetermined etiology | Allogeneic HSCT | CNS infections predominantly early (<4 months); bacterial CNS infection rare | Eleven patients died (73%), 7 (47%) directly of the CNS infection |
| Autopsy-based study | | | | | | | | | |
| Bleggi-Torres, 2000 (Brazil) ^1^ | Autopsy study | 180 BMT autopsies (mostly allogeneic) | Neuropathological findings | CNS infection in 15% of autopsies | At least 13% (24/180): 16 fungal: 8 *Aspergillus spp.*, 5 *Candida spp.*, 1 *Fusarium spp.*, 2 other fungi; 8 Toxoplasma | 3 bacterial CNS infections, unclear if abscess or meningitis: 2 *S. epidermidis*, 1 *S. aureus* | Not applicable | Autopsy based study | CNS pathology cause of death in 31 (17%): 8/16 with fungal infection and 3/8 with toxoplasmosis |
| Only paediatric population | | | | | | | | | |
| Zaidman, 2022  (Israel) ^38^ | Multicentre, retrospective | 746 patients underwent 943 allo-HSCT | Incidence, risk factors, and outcomes of NC after paediatric HSCT | 3.8% (28/746) infectious CNS complications | 1.4% (11/746): 8 toxoplasmosis, 3 fungal (1 *Aspergillus*, 1 *Candida*, 1 *Cryptococcus*); in addition, ,  3 *Mycobacterium tuberculosis;* not clear if clinical presentation was abscess or meningitis, counted with meningitis | 7 (1%); 4 pneumococcus, 3 *M. tuberculosis* | Children with immunodeficiencies experienced mostly infectious NCs | Infectious NC significantly associated with developing neurological sequela | Type of NC was significantly correlated with the cause of death: 58.8% of the infectious group having a neurological cause of death vs. 26.2% of the non-infectious group |
| Weber, 2008 (Germany) ^22^ | Single-centre retrospective; | 165 paediatric HSCT recipients (111 allo-HSCT, 54 auto-HSCT) | NC after paediatric HSCT | 5.4% (9/165 infectious CNS complications) | 3% (5/165): 3 toxoplasmosis, 2 Aspergillus spp. | 0 bacterial meningitis cases reported | Unrelated allogeneic HSCT; prolonged neutropenia; GVHD | Detailed paediatric cohort; infections only among patients with progressive neurological symptoms | All 5 patients with toxoplasmosis or IFD died. |
| Uckan, 2005 (Turkey) ^76^ | Single-centre retrospective; | 113 paediatric allo-HSCT recipients | NC after HSCT | 1.8% (2/113 intracranial infections) | Included within 2 cases of brain abscess / meningoencephalitis; etiology not specified | 0 bacterial meningitis cases reported | Not reported for CNS infection | Only severe and life-threatening neurological events included; minor infections excluded | Very high mortality: 10/11 patients with severe neurological complications died |
| Faraci, 2002 (Italy) ^77^ | Single-centre retrospective; | 272 paediatric HSCT recipients (115 related allo-HSCT, 70 unrelated allo-HSCT, 87 auto-HSCT) | Incidence and risk factors of severe neurologic events after HSCT | 2.6% (7/272) | 0.4%: 1 fungal abscess (Aspergillus spp.) | 0 bacterial meningitis cases reported | Unrelated donor HSCT; acute GVHD ≥ grade II; TBI | Only severe (grade 3–4) neurologic events included; no bacterial CNS infections observed | CNS infection mortality 57% (4/7 infection-related deaths) |
| Autopsy-based study, including both adult and paediatric patients | | | | | | | | | |
| Bleggi-Torres, 2000 (Brazil) ^1^ | Autopsy study | 180 BMT autopsies (mostly allogeneic) | Neuropathological findings | CNS infection in 15% of autopsies | At least 13% (24/180): 16 fungal: 8 *Aspergillus spp.*, 5 *Candida spp.*, 1 *Fusarium spp.*, 2 other fungi; 8 Toxoplasma | 3 bacterial CNS infections, unclear if abscess or meningitis: 2 *S. epidermidis*, 1 *S. aureus* | Not applicable | Autopsy based study | CNS pathology cause of death in 31 (17%): 8/16 with fungal infection and 3/8 with toxoplasmosis |

 Allo-HSCT, allogeneic haematopoietic cell transplantation; auto-HSCT, autologous haematopoietic cell transplantation; CMV, cytomegalovirus; CNS, central nervous system; EBMT, European Society for Blood and Marrow Transplantation; EBV, Epstein–Barr virus; GvHD, graft-versus-host disease; HSCT, haematopoietic cell transplantation; HHV-6, human herpesvirus 6; HSV, herpes simplex virus; IFD, invasive fungal disease; JCV, John Cunningham virus; NC, neurological complications; ND, not determined/not described; NRM, non-relapse mortality; OS, overall survival; PFS, progression-free survival; TBI, total body irradiation; UCBT, umbilical cord blood transplantation; VZV, varicella-zoster virus.

## **Table S2.**

Studies on bacterial meningitis after HSCT and on meningeal involvement in HSCT recipients with invasive pneumococcal disease.

| Reference (first author, year, country) | Study design, population | Study population (Denominator issue) | Study main endpoint | Rate of bacterial meningitis; detailed etiology | Risk factors | Comments | Outcome |
| --- | --- | --- | --- | --- | --- | --- | --- |
| Bacterial meningitis | | | | | | | |
| Oyama, 2021 (Japan) ^78^ | Single-centre retrospective; allo-HSCT (CBT focus) | 1465 allo-HSCT (1147 CBT); 7 bacterial meningitis cases, all CBT | Clinical characteristics of bacterial meningitis after CBT | Pathogens: 2 *Enterococcus feacium*, 1 *E. gallinarum,* 1 *Staphylococcus haemolyticus*, 1 *Streptococcus mitis*, 1 *Rothia* | Cord blood transplant; pre-engraftment phase; **all had concomitant BSI**, time from BSI to meningitis: median 10 days (range, 2-27) | Reports early post-HSCT (between days 5 and 45), all non-pneumococcal; 5/7 patients developed meningitis before neutrophil engraftment | High mortality (5/7) |
| Glimåker, 2020 (Sweden) ^42^ | National ABM quality registry | 1056 adult ABM cases; 352 immunocompromised (HSCT not separated) | Compare ABM in immunocompromised vs immunocompetent adults | Not HSCT-specific; in immunocompromised ABM: *S. pneumoniae* 55%, *Listeria 1*4%, Alfa-haemolytic streptococci 5%, Beta-haemolytic streptococci 6%, S. aureus 5%, Enterobacterales 3%, *H. influenzae* 3% | Immunocompromised state | Comparator study only; no HSCT-specific incidence | Mortality modestly higher in immunocompromised |
| van Veen, 2016 (Netherlands) ^43^ | Nationwide prospective cohort of ABM | 14 HSCT-associated ABM episodes; population-based denominator | Presentation and outcome of bacterial meningitis in HSCT | 11 *S. pneumoniae*, 2 *N. meningitidis*, 1 *S. mitis* | Allogeneic HSCT; late post-transplant period:  median interval 4 years (range 4 days-13 years), but 13/14 patients had late onset (>100 d); 1 patient 4 days after HSCT | 70/100 000/y allo-HSCT (95% CI 23.9–62.2); 15.8/100 000/y auto-HSCT (95% CI 5.1–49.2)  Incidence in HSCT 30-fold (95% CI 18–51) higher compared with persons without HSCT: 52-fold (95% CI 29–94, P < 0.001) higher for allo-HSCT recipients and 12-fold (95% CI 4–36; P<0.001) higher for autologous HSCT | 21% mortality; 55% neurologic sequelae |
| Invasive pneumococcal disease | | | | | | | |
| Okinaka, 2023 (Japan) ^79^ | Nationwide retrospective registry | 22,514 allo-HSCT recipients ≥100 days; 43 patients, 49 pneumococcal episodes | Clinical spectrum and risk factors of late pneumococcal disease after allo-HSCT | 3 pneumococcal meningitis episodes (late onset) | Chronic GVHD; viral infection; complete remission at HSCT | Largest late-phase allo-HSCT pneumococcal cohort | 14% mortality; rapid death (<7 days) common |
| Olarte, 2017 (USA, paediatric) ^80^ | Multicentre prospective surveillance (PCV era) | 21 paediatric HSCT recipients with IPD (8 centres); HSCT denominator not available | Epidemiology of IPD in paediatric transplant recipients | 0 pneumococcal meningitis in HSCT recipients | Low PCV/PPSV23 uptake; late post-transplant period | First PCV-era paediatric HSCT IPD study | No IPD-attributable deaths, but recurrent IPD in 10% |
| Torda, 2014 (Australia) ^81^ | Multicentre retrospective case–control | 1154 adult allo-HSCT recipients; 26 IPD episodes in 23 patients | Incidence and risk factors of IPD after allo-HSCT | 1 pneumococcal meningitis episode (3.8% of IPD) | Hyposplenism/asplenia; MMF; chronic GVHD; relapse | High-quality risk-factor modelling | 30% IPD-attributable mortality |
| Debbache, 2009 (France) ^82^ | Two-centre retrospective microbiology study | 22 haematology patients with IPD (9 allo-HSCT, 8 auto-HSCT); denominator not population-based | Pneumococcal serotype coverage by vaccines | 0 pneumococcal meningitis cases | GVHD; steroid exposure | Serotype-focused; not an incidence study | 3 deaths, all recurrent IPD |
| Kumar, 2008 (Canada) ^83^ | Prospective population-based surveillance | 1238 adult HSCT recipients (431 allo-HSCT, 807 auto-HSCT); 14 IPD cases | Incidence, serotypes, resistance of IPD after HSCT | 0 pneumococcal meningitis cases reported | Chronic GVHD (significant) | Population surveillance; Any pneumococcal infections: HSCT 347/ 100 000 persons per year vs. 11.5 per 100 000 persons per year in the general population (30-fold higher rate) | 14% in-hospital mortality |
| Engelhard, 2002 (Europe) ^84^ | Prospective multicentre EBMT survey | 8547 HSCT recipients; 51 IPD episodes (early + late) | Incidence, risk factors, and outcome of IPD after HSCT | 10 pneumococcal meningitis episodes (all late ≥100 days), overall rate 0.2% | Allogeneic HSCT; chronic GVHD; steroids; hypogammaglobulinemia; hyposplenism | Landmark EBMT pneumococcal study | 20% overall mortality; 30% meningitis mortality |

ABM: acute bacterial meningitis; allo-HSCT: allogeneic hematopoietic cell transplantation; auto-HSCT: autologous hematopoietic cell transplantation; BSI: bloodstream infection; CBT: cord blood transplantation; CI: confidence interval; EBMT: European Society for Blood and Marrow Transplantation; GVHD: graft-versus-host disease; H. influenzae: Haemophilus influenzae; HSCT: hematopoietic cell transplantation; IPD: invasive pneumococcal disease; MMF: mycophenolate mofetil; N. meningitidis: Neisseria meningitidis;; PCV: pneumococcal conjugate vaccine; PPSV23: 23-valent pneumococcal polysaccharide vaccine.

## **Table S3.**

Studies on fungal focal central nervous system (CNS) infections (excluding case reports and small case series).

| Reference: first author, year (country) | Study design, period | Patient population (n) | Diagnostic certainty; year of EORTC criteria used, if applicable^*^ | Pathogen | Extra-CNS infection | Treatment | Surgery | Mortality |
| --- | --- | --- | --- | --- | --- | --- | --- | --- |
| Karaman, 2022 (Turkey) ^85^ | Multicentre  Retrospective  2005-2015 | Paediatric HM  (51; 6/51 HSCT) | Proven (17, 33%), probable (34, 67%); 2020 | *Aspergillus* spp. (8), *Candida* spp. (7), Mucorales (2), *Trichoderma* (2), Other (3) | 56%  (28, lung) | Monotherapy 31% (AMB, 13; VCZ, 3)  Combination 69% (AMB-VCZ, 22; AMB-PCZ, 4; AMB-CAS, 4; other, 5) | 45% (23) | 43% |
| Candoni, 2020 (Italy) ^86^ | Multicentre  Retrospective  2010-2016 | Paediatric/Adult allo-HSCT (24) | Proven (14, 58%), probable (10, 42%); 2008 | *Aspergillus* spp. (17), Mucorales (3), *Fusarium* spp. (1), *Scedosporium* spp. (1), *Cryptococcus* spp. (1), *Trichosporon* spp. (1) | 75% (18) | Monotherapy 66.7% (16)  Combination 33.3% (8)  LAMB 14, VCZ 11 | 17% (4) | 75% |
| Lauten, 2019 (Germany) ^873^ | Multicentre  Retrospective  2007-2016 | Paediatric HM  (29; 14/29 allo-HSCT) | Proven (12, 41%), probable (17, 59%); 2008 | *Aspergillus* spp. (14), Mucorales (3), *Fusarium* spp. (1) | 96.6% (28) | Monotherapy 41.7% (10)  Combination 58.3% (14)  VCZ 19, LAMB 15 | 44.8% (13) | 34.5% (30-day), 51.1% (2-year) |
| Economides, 2017 (USA) ^88^ | Single-centre  Retrospective  2001-2016 | Adult HM  (40; 17/40 allo-HSCT) | Proven (16, 40%), probable (24, 60%); 2008 | *Aspergillus* spp. (13), Mucorales (9), *Curvularia* (1) | 90% (36, lung) | Monotherapy 18% (7)  Combination therapy 82% (33: LAMB-VCZ, 25; LAMB-ECH, 20; LAMB-PCZ, 11) | 43% (17) | 48% (42-day) |
| Balaguer, 2017 (Spain) ^89^ | Single-centre  Retrospective  2000-2014 | Adult HSCT  (12; allo-HSCT 11, CB 1) | Proven (8, 66.7%), probable (4, 33.3%); 2008 | *Aspergillus* spp. (8), Mucorales (1), *Scedosporium prolificans* (1), *Cryptococcus neoformans* (2) | NS | NS | NS | 92% |
| Sun, 2017 (China) ^39^ | Single-centre  Retrospective  2007-2016 | Paediatric/Adult allo-HSCT (29) | Proven (5, 17.2%), probable (21, 72.4%), possible (3, 10.3%); 2008 | *Aspergillus* spp. (9), *Rhizopus* (1), *Scedosporium apiospermum* (1) | 83% (23, lung) | Monotherapy 83% (24: VCZ)  Combination 17% (5: AMB-VCZ) | 0 | 51.8% (12-weeks), 76% (overall) |
| Jantunen, 2003 (Norway) ^90^ | Single-centre  Retrospective  1997-2003 | Adult allo-HSCT (14) | Proven (14, 100%) | *Aspergillus* spp. (14) | NS | AMB 86% (12) | NS | 100% |
| Baddley, 2002 (USA) ^91^ | Single-centre  Retrospective  1997-2000 | Paediatric/Adult allo-HSCT (12) | NS | *Aspergillus* spp. (8), *Scedosporium* spp. (2), *Microascus cinereus* (1), *Mucor* spp. (1) | 75% (9) | NS | NS | 100% |

CB, cord blood; IFI, invasive fungal infection, CNS, central nervous system, HM, hematologic malignancy, HSCT, hematopoietic cell transplant, AMB, amphotericin-B, VCZ, voriconazole, PCZ, Posaconazole, CAS, caspofungin, LAMB, liposomal AMB, ECH, echinocandin, NS, not specified.

^*^Diagnosis made based on IFI criteria published in 2020 ^92^ or 2008 ^93^.

Karaman, 2022 (Turkey) ^85^ : Patients from 23 cancer/bone marrow transplant centres were reported. All patients with HM were diagnosed with leukaemia. There were only 22 cases diagnosed based on a positive culture result. A biopsy was performed in 17/71 (33.3%) of patients.

Candoni, 2020 (Italy) ^86^ :  A total of 3866 allogeneic HSCT recipients were included from 13 bone marrow transplant centres, with an estimated incidence of CNS IFI of 0.6%. A biopsy was performed in 8% of cases.

Lauten, 2019 (Germany) ^873^ : There were 14 cases of *A. fumigatus* and amongst patients with Mucorales IFI the following pathogens were identified: *Rhizopus arrhizus, Absidia corymbifera, Rhizomucor pussilus*. In one patient two pathogens were identified by culture. There were 12 patients with IFI with negative cultures and PCR results.

Economides, 2017 (USA) ^88^ : The overall incidence was 3.8 cases / 100,000 patient-days. There were only 23/40 patients with IFI with a microbiological diagnosis. Pathology results were available in 16/40 (40%) cases, 10 and 6 of which were performed pre- and post-mortem, respectively.

Balaguer, 2017 (Spain) ^89^ : This cohort included patients with non-fungal CNS infections, but there were 12 IFI identified. No specific information provided on treatment.

Sun, 2017 (China) ^39^ : A total of 3855 HSCT recipients were included during the study period, with an overall incidence of CNS fungal infections of 0.8%. A microbiological diagnosis was obtained in only 11/29 (38%) of cases. A pathogen was identified on a brain biopsy in 5 cases.

Jantunen, 2003 (Norway) ^90^ : Only allogeneic HSCT recipients with CNS infections due to *Aspergillus* spp. were included. In a cohort of 455 patients, there were 14 cases identified (incidence 3%), all diagnosed at autopsy.

Baddley, 2002 (USA) ^91^ : Review of all fungal CNS infections in solid organ transplant and HSCT paediatric (>=15) and adult (>=18-year-old) recipients. A total of 12 cases of fungal CNS infections were identified amongst 128 allogeneic HSCT (incidence 9.4%).

## **Table S4.**

Studies on CNS toxoplasmosis after HSCT (case reports excluded).

| Study | Study design | Patients Population (N) | CNS toxoplasmosis cases | Rate, % | Timing after HSCT | CSF / microbiology | Prophylaxis at event | Outcome |
| --- | --- | --- | --- | --- | --- | --- | --- | --- |
| Malek et al., 2024 ^94^ | Single-centre retrospective | Adults, allo-HSCT n=314 | 11 | 3.50 | Median 38 days (15–202) | PCR in blood and CSF in CNS cases | Some on atovaquone; many without active prophylaxis | 5 deaths |
| Xhaard et al., 2023 ^95^ | Multicentre cohort | Adults, 1258 allo-HSCT | 7 | 0.56 | N/A | PCR in CSF | Not detailed | Not detailed for CNS |
| Aerts et al., 2022 ^96^ | Single-centre retrospective cohort | Adults, 1455 allo- HSCT | 16 | 1.10 | Median 45 days (13–140 days) | N/A | TMP-SMX if PCR positive | Not detailed |
| Paccoud et al., 2020 ^97^ | Retrospective cohort (high seroprevalence area) | Adults, 318 allo-HSCT | 6 | 1.89 | N/A | PCR in CSF | Variable; some without prophylaxis | 1 death for CNS |
| Czyżewski et al., 2019 ^98^ | Multicentre national cohort | Paediatric, allo-HSCT 287 | 3 | 1.05 | Day +58, +90, +352 | PCR in blood, CSF, vitreous fluid | TMP-SMX | 0 deaths |
| Chueng et al., 2019 ^99^ | Retrospective cohort | Adults, HSCT 126 (94 allo-HSCT, 23 auto-HSCT) | 2 | 1.59 | Median 66 days (52–67) | PCR in CSF and/or blood | Atovaquone | 1/10 survived |
| Robin et al., 2019 ^100^ | Multicentre retrospective cohort | Adults, 419 allo-HSCT | 2 | 0.48 | N/A | N/A | Not detailed | Not detailed for CNS |
| Balaguer-Roselló et al., 2017 ^71^ | Single-centre retrospective cohort | Adults, 366 MSD + 343 UCB allo-HSCT. | 4 | 0.56 | N/A | PCR in CSF | TMP-SMX for PCP prophylaxis (twice weekly; possibly suboptimal for toxoplasmosis) | Not detailed for Toxoplasma |
| Decembrino et al., 2017 ^101^ | Retrospective cohort | Paediatric, allo-HSCT 187 | 3 | 1.60 | N/A | PCR in CSF and/or blood | TMP-SMX or pentamidine | 2 deaths |
| Conrad et al., 2016 ^102^ | Matched case-control | Adults, allo-HSCT 588 | 10 | 1.70 | N/A | N/A | 82.6% without effective prophylaxis | Not detailed for CNS |
| Hanajiri et al., 2016 ^70^ | Retrospective cohort of CNS infections | Adults, 353 allo-HSCT | 1 | 0.28 | Day 101 | PCR in CSF | Not detailed | Fatal |
| Mendorf et al., 2015 ^103^ | Retrospective cohort | Adults, 155 allo-HSCT | 2 | 1.29 | N/A | N/A | Not detailed | Not detailed for CNS |
| Hakko et al., 2013 ^104^ | Single-centre retrospective | Adults, 170 allo-HSCT | 5 | 2.94 | Median 84 days (51–184) | CSF: mild ↑ protein; PCR in CSF in 4/5 | Only 1/5 on TMP-SMX | 2 deaths |
| Sumi et al., 2013 ^105^ | Retrospective single-centre | Adults, 277 HSCT (87 auto-HSCT, 192 allo-HSCT) | 2 (auto-HSCT + allo-HSCT) | 0.72 | N/A | N/A | Not detailed | Not detailed for CNS |
| Mulanovic et al., 2011 ^106^ | Retrospective cohort | Adults, 168 allo-HSCT | 6 | 3.57 | Median 64 days (range, 0–237 days | PCR in CSF | Not detailed | 5 deaths |
| Meers et al., 2010 ^107^ | Prospective monitoring program analysed retrospectively | Adults and paediatrics, 208 allo-HSCT | 5 | 2.40 | NA/ | PCR in CSF | With monitoring and pre-emptive therapy, low toxoplasma-related mortality reported (3 deaths) in cohort | 3 deaths |
| Weber et al., 2008 ^22^ | Retrospective paediatric cohort | Paediatrics, 165 allo-HSCT | 3 | 1.82 | N/A | N/A | Not detailed | 3 deaths |
| Denier et al., 2006 ^20^ | Retrospective cohort of neurologic complications after HSCT | Adults, 361 (245 auto-HSCT, 116 allo-HSCT) | 5 | 1.39 | N/A | CSF: ↑ protein (≈0.65–1.0 g/L), variable pleocytosis; diagnostic yield of serology/PCR limited | Not detailed | One death |
| Roemer et al., 2001 ^108^ | Retrospective single-centre | Adults, 301 allo-HSCT | 8 | 2.66 | Median 166 (90–280 days | PCR in CSF | Not detailed | 5 deaths |
| Martino et al., 2005 ^109^ | Prospective study European | Adults, allo-HSCT 106 (all seropositive) | 4 | 3.77 | N/A | PCR in CSF | Not detailed | 1 death |

allo-HSCT: allogeneic hematopoietic cell transplantation; auto-HSCT: autologous hematopoietic cell transplantation; HSCT: hematopoietic cell transplantation; CNS: central nervous system; CSF: cerebrospinal fluid; PCR: polymerase chain reaction; TMP-SMX: trimethoprim–sulfamethoxazole; PCP: *Pneumocystis* pneumonia (*Pneumocystis jirovecii* pneumonia); MSD: matched sibling donor; UCB: umbilical cord blood; EBMT: European Society for Blood and Marrow Transplantation; N/A: not available / not applicable.

## **Table S5.**

Cases of CNS *Acanthamoeba* infections in HSCT recipients, all single patient case reports

| Study | Patient population (N) | Timing after HSCT | Neuroimaging | CSF / Microbiology | Prophylaxis at event | Treatment | Outcome |
| --- | --- | --- | --- | --- | --- | --- | --- |
| Al Dhaheri et al., 2023 ^110^ | Adult allo-HSCT | Day +150 | MRI: frontal lobe lesions | Skin biopsy PCR positive; CNS involvement radiologic | Voriconazole | Miltefosine, fluconazole, voriconazole, flucytosine, pentamidine, azithromycin, sulfadiazine | response; later death (non-GAE) |
| Keane et al., 2020 ^111^ | Adult allo-HSCT | 5 months | MRI: multiple circumscribed heterogeneous mass-like lesions | Brain biopsy: cysts /trophozoites; PCR positive | Atovaquone | Miltefosine, azole, flucytosine, pentamidine, cotrimoxazole | Survived |
| Matsui et al., 2018 ^112^ | Adult allo-HSCT | 15 months | MRI: parietal–occipital mass lesions | Brain autopsy, PCR (genotype T18) | Not specified | None (post-mortem diagnosis) | Death |
| Coven et al., 2017 ^113^ | Paediatric allo-HSCT | Day +226 | MRI oedema, non specific | CSF non-diagnostic; | Not specified | None (post-mortem diagnosis) | Death |
| Doan et al., 2015 ^114^ | Adult auto-HSCT | Day +15 | CT/MRI: multifocal vasogenic oedema, patchy enhancement | Brain biopsy positive for *Acanthamoeba* | Not specified | None (post-mortem diagnosis) | Death |
| Abd et al., 2009 ^112^ | Adult allo-HSCT | First symptoms after 2 weeks | Not detailed | CSF microscopy, culture, PCR positive for *Acanthamoeba castellanii*, brain biopsy positive | Not specified | None (post-mortem diagnosis) | Death |
| Kaul et al., 2008 ^115^ | Adult allo-HSCT | Day +930 | MRI: mass-like brain lesions | Skin biopsy diagnostic | Not specified | Multidrug (azole, macrolide, pentamidine, sulfadiazine) | Death |
| Pemán et al., 2008 ^28^ | Adult allo-HSTC | Day +90 | MRI negative then oedema and herniation | CSF negative (diagnosis in autopsy) | TMP-SMX, fluconazole | None (post-mortem diagnosis) | Death |
| Castellano-Sanchez et al., 2004 ^116^ | Adult allo-HSCT | Day +140 | CT/MRI: multifocal mass-like lesions (posterior fossa, parietal–occipital lobes) | Diagnosis at autopsy (brain & lung tissue); immunofluorescence positive for *Acanthamoeba* | TMP-SMX, itraconazole, inhaled pentamidine | None (post-mortem diagnosis) | Death |

allo-HSCT: allogeneic hematopoietic cell transplantation; allo-HSTC: allogeneic hematopoietic stem cell transplantation (likely typographical variant of allo-HSCT); auto-HSCT: autologous hematopoietic cell transplantation; HSCT: hematopoietic cell transplantation; CNS: central nervous system; CT: computed tomography; MRI: magnetic resonance imaging; CSF: cerebrospinal fluid; PCR: polymerase chain reaction; TMP-SMX: trimethoprim–sulfamethoxazole; GAE: granulomatous amoebic encephalitis.

## **Table S6.**

Targeted therapies for CNS infection due to selected main pathogens relevant in HSCT recipients

| Pathogen | Antimicrobial regimen | Comments | References |
| --- | --- | --- | --- |
| Bacterial brain abscess | Antibiotics should be selected based on the results and susceptibility testing, if available  Most frequently used for bacterial abscess are ceftriaxone 2 g q12h or cefotaxime 2 g q4–6h plus metronidazole 500mg q8h in the general population  Vancomycin (15-20 mg q8-12h), or in alternative linezolid (600 mg q12h), in case of resistant streptococci or MRSA | - Together with clinical experience and susceptibility results consider also CNS penetration. Drugs with good CNS penetration should be preferred   - Good CNS penetration even without CNS inflammation: fluoroquinolones, linezolid, metronidazole, TMP-SMX   - Limited CNS penetration but enhanced by inflammation: penicillins, 3^rd^/4^th^ generation cephalosporins (e.g., ceftriaxone, cefepime), carbapenems, vancomycin.   - Poor CNS penetration: aminoglycosides, macrolides, clindamycin, 1^st^ and 2^nd^ generation cephalosporins, vancomycin if no inflammation. - The strategies to increase CNS penetration include high dose IV therapy or particularly used in case of ventriculitis due to resistant bacteria, intrathecal administration | ^117,118^ |
| Aspergillosis^*^ | Voriconazole IV 6 mg/kg q12h for 1 day and 4 mg/kg q12h hours thereafter  Or  Isavuconazole 200 mg q8h for 2 days and 200 mg/day thereafter  Or  L-AmB 5 mg/kg/day in patients with azole- resistant isolates or in case of azole intolerance | - Voriconazole is proposed based on international guidelines recommendations and its excellent bioavailability and penetration into the CNS - When treating CNS infections with a broad-spectrum azole, TDM is strongly recommended - Isavuconazole was shown non inferior to voriconazole for invasive aspergillosis, adequate penetration into infected brain tissue has been reported - Minimal treatment duration of 3 months is usually recommended. Duration may also depend on the administration of immunosuppression and patient immune reconstitution - Treatment should be driven by susceptibility testing, if available, or the probability of azole-resistant infection | ^119-124^ |
| Mucormycosis^*^ | L-AmB 5-7.5mg/kg/day  Alternative treatment options may include (particularly as step-down treatment):  Isavuconazole 200 mg q8h for 2 days  and 200 mg/day thereafter  Or  Posaconazole 300 mg q12 h for 1 day and 300 mg q24 hours thereafter | - As higher doses of L-AmB are associated with higher nephrotoxicity and discontinuation rates, doses between 5 and 7.5 mg/kg/day may be preferable to 10 mg/kg/day to attain efficacy without compromising safety - When treating CNS infections with a broad-spectrum azole, TDM is strongly recommended - The role of combination antifungal therapy in the management of CNS IFI remains unclear - Treatment duration of 3-6 months of treatment is usually recommended. Duration may also depend on the administration of immunosuppression and immune reconstitution of the patient | ^125-130^ |
| Toxoplasmosis | Pyrimethamine (200 mg single loading dose followed by 50–75 mg/day orally) plus folinic acid (10–25 mg/day) in combination with a second active agent:   - sulfadiazine 4–6 g/day - clindamycin 600 mg q6h (orally or IV), in patients intolerant to sulfadiazine - atovaquone (1500 mg/day orally) if intolerant to other regimens   Alternative treatment options:  TMP-SMX (15 mg of TMP/day) with or without clindamycin (600 mg q6-8h) [if pyrimethamine is not available or oral route is not feasible]  Atovaquone in combination with sulfadiazine (daily doses above) | - Minimum duration of therapy:   - 6 weeks or until clinical resolution, or both   - 2 negative PCR tests in blood, 7 days apart, or one qPCR negative in a previously qPCR- positive cerebrospinal fluid - Longer courses if clinical disease or radiological findings are extensive or response is incomplete at 6 weeks - Immunosuppressive therapy lowering or discontinuation is recommended, when possible - Steroids to be considered in patients with severe ocular toxoplasmosis or CNS disease with radiological midline shift, progression within 48 h of treatment or elevated intracranial pressure. | ^13^ |
| Nocardiosis | A combination of TMP-SMX with imipenem (500mg q6h), with or without high-dose amikacin (20mg/kg/day)  Alternative: linezolid and imipenem | - Initial IV combination provided for 2-6 weeks can be followed by oral monotherapy preferably with TMP/SMX, if tolerated, based on in-vitro susceptibility and clinical and radiological response, with a total duration of at least 9-12 months - Surgical drainage should be considered in patients with large abscesses, clinical deterioration, insufficient response after 3-4 weeks of therapy, on suspicion on another infection, or on suspicion of potential complications as presence of mass effect with brain shift and pending herniation or proximity to the ventricles with the risk of abscess rupture into the ventricular system. Limited evidence demonstrates better outcome with surgical intervention | ^6,11,131-133^ |
| Listeriosis | IV ampicillin (2 g q4h) or amoxicillin or penicillin G; cotrimoxazole for patients with penicillin allergies; meropenem is another alternative. | - Adding gentamicin to ampicillin or penicillin is controversial. The rationale for combination therapy includes achieving in-vitro synergism and counteracting the delayed bactericidal activity in the CSF. Clinical studies show conflicting results: supporting combination in some ^51,134^, nor reporting difference in others ^135-137^ ^138^ ^139^, and reporting worse outcome in some ^140,141^ - Treatment duration is generally three weeks for meningitis and four to six weeks for abscesses - Adjunctive steroid administration is controversial (see text for details) | ^51,134-142^ |

^*^Prompt surgical intervention for debridement and resection of lesions is strongly recommended in patients with CNS mould infection, particularly due to *Mucorales*, when feasible and accessible ^126^.

CNS: central nervous system; MRSA: methicillin-resistant Staphylococcus aureus; q12h: every 12 hours; q8h: every 8 hours; q24h: every 24 hours; IV: intravenous; mg: milligram; g: gram; kg: kilogram; mg/kg: milligrams per kilogram; L-AmB: liposomal amphotericin B; IFI: invasive fungal infection; PCR: polymerase chain reaction; qPCR: quantitative polymerase chain reaction; SMX: sulfamethoxazole; TDM: therapeutic drug monitoring; TMP-SMX: trimethoprim–sulfamethoxazole; CSF: cerebrospinal fluid.

## **Table S7.**

Paediatric doses for empirical and targeted treatment of CNS infections.

| Antibiotics | Dose |
| --- | --- |
| Ampicillin | 50 - 65 mg/kg/dose every 4 hours (> 1 month) |
| Cefepime | 50 mg/kg/dose every 8 hours (≥ 2 months) |
| Cefotaxime | 55-75 mg/kg/dose every 6 hours (>1mo) |
| Ceftazidime | 50 - 65 mg/kg/dose every 8 hours |
| Ceftriaxone | 50 mg/kg/dose every 12 hours (max 2 g) (> 1 month) |
| Imipenem-cilastatin | 15-25 mg/kg/dose every 6 h (≥3 months) |
| Meropenem high dose | 40 mg/kg/dose (max 2 g) every 8 hours (> 1 month) |
| TMP-SMX high dose | 15-20 mg/kg/day of TMP component (≥ 2 months) |
| Vancomycin | 15-20 mg/kg/dose every 6 hours (> 1 month) |
| L-AmB | 5 mg/kg/day |
| Isavuconazole IV | 3-18 years and ≤37 kg: 5.4 mg/kg/day (max 200 mg) (5.4 mg/kg every 8 hours on day 1 and day 2) |
| Voriconazole (follow with therapeutic drug monitoring) | 2–12 years or 12–14 years and < 50 kg: 8 mg/kg every 12 hours (9 mg/kg every 12 hours on day 1)  12–14 years and ≥ 50 kg or more, or ≥ 15 years: 4 mg/kg every 12 hours (6 mg/kg every 12 hours on day 1) |

L-AmB, liposomal amphotericin B; TMP-SMX, trimethoprim-sulfamethoxazole

## **Table S8.**

Summary of Key EBMT Consensus Recommendations.

| Area | Consensus Recommendations |
| --- | --- |
| Aetiology | Non-viral CNS infections after HSCT include focal brain lesions and meningitis due to bacterial, fungal and parasitic (mainly *Toxoplasma* spp.) pathogens. |
| Definitions | Definitions of CNS infections, such as focal brain lesions of infectious origin and meningitis, include clinical, radiological and laboratory findings, based on which the diagnostic certainty of proven and probable infectious origin is established. |
| Diagnosis | Diagnostic work-up in patients with suspected brain focal lesions and meningitis include clinical suspicion, neuroimaging, CNS and non-CNS microbiological tests, evaluation of extra-CNS sites, and ultimately an invasive diagnostic procedure, such as brain biopsy, if feasible. |
| Empirical therapy | Empirical therapy of focal brain lesions should target common bacterial pathogens, *Nocardia* spp., and toxoplasmosis. Antifungal empirical treatment should be considered based on clinical and radiographical suspicion. |
|  | Empirical therapy of meningitis should include coverage of common bacterial pathogens and *Listeria monocytogenes*. Administration of corticosteroids should be considered when S. pneumoniae and H. influenzae are likely causes of meningitis. |
| Response assessment | Response should be assessed in 6-12 weeks and 2-4 weeks in patients with focal lesions and meningitis, respectively and can be defined as success or failure. Success includes complete or partial response whereas failure includes stable disease, progression, and/or death. Neurological sequalae should be reported separately from the overall response assessment. |

# **References**

1.	Bleggi-Torres LF, de Medeiros BC, Werner B, et al. Neuropathological findings after bone marrow transplantation: an autopsy study of 180 cases. *Bone Marrow Transplant* 2000; **25**(3): 301–7.

2.	van Burik JA, Hackman RC, Nadeem SQ, et al. Nocardiosis after bone marrow transplantation: a retrospective study. *Clin Infect Dis* 1997; **24**(6): 1154–60.

3.	Kurosawa S, Sekiya N, Doki N, et al. The emergence of rare nocardiosis following allogeneic hematopoietic stem cell transplantation in the era of molecular taxonomy. *Int J Infect Dis* 2019; **89**: 154–62.

4.	Daly AS, McGeer A, Lipton JH. Systemic nocardiosis following allogeneic bone marrow transplantation. *Transpl Infect Dis* 2003; **5**(1): 16–20.

5.	Averbuch D, De Greef J, Duréault A, et al. Nocardia Infections in Hematopoietic Cell Transplant Recipients: A Multicenter International Retrospective Study of the Infectious Diseases Working Party of the European Society for Blood and Marrow Transplantation. *Clin Infect Dis* 2022; **75**(1): 88–97.

6.	Yetmar ZA, Marty PK, Clement J, Miranda C, Wengenack NL, Beam E. State-of-the-Art Review: Modern Approach to Nocardiosis-Diagnosis, Management, and Uncertainties. *Clin Infect Dis* 2025; **80**(4): e53–e64.

7.	De Greef J, Averbuch D, Tondeur L, et al. Risk factors for Nocardia infection among allogeneic hematopoietic cell transplant recipients: A case-control study of the Infectious Diseases Working Party of the European Society for Blood and Marrow Transplantation. *J Infect* 2024; **88**(6): 106162.

8.	Roussel X, Daguindau E, Berceanu A, et al. Altered thymic CD4(+) T-cell recovery after allogeneic hematopoietic stem cell transplantation is critical for nocardiosis. *Curr Res Transl Med* 2019; **67**(4): 135–43.

9.	Molina A, Winston DJ, Pan D, Schiller GJ. Increased Incidence of Nocardial Infections in an Era of Atovaquone Prophylaxis in Allogeneic Hematopoietic Stem Cell Transplant Recipients. *Biol Blood Marrow Transplant* 2018; **24**(8): 1715–20.

10.	Gkirkas K, Stamouli M, Thomopoulos T, et al. Low-Dose Cotrimoxazole Administered in Hematopoietic Stem Cell Transplant Recipients as Prophylaxis for Pneumocystis jirovecii Pneumonia Is Effective in Prevention of Infection due to Nocardia. *Biol Blood Marrow Transplant* 2019; **25**(9): e298–e9.

11.	Lebeaux D, Coussement J, Bodilsen J, Tattevin P. Management dilemmas in Nocardia brain infection. *Current opinion in infectious diseases* 2021; **34**(6): 611–8.

12.	Corsini Campioli C, Castillo Almeida NE, O'Horo JC, et al. Clinical Presentation, Management, and Outcomes of Patients With Brain Abscess due to Nocardia Species. *Open Forum Infect Dis* 2021; **8**(4): ofab067.

13.	Aerts R, Mehra V, Groll AH, et al. Guidelines for the management of Toxoplasma gondii infection and disease in patients with haematological malignancies and after haematopoietic stem-cell transplantation: guidelines from the 9th European Conference on Infections in Leukaemia, 2022. *Lancet Infect Dis* 2024; **24**(5): e291–e306.

14.	Malek AE, Al-Juhaishi T, Milton DR, et al. Outcomes of toxoplasmosis after allogeneic hematopoietic stem cell transplantation and the role of antimicrobial prophylaxis.  Bone Marrow Transplantation: Springer Nature; 2024. p. 699–704.

15.	Czyzewski K, Fraczkiewicz J, Salamonowicz M, et al. Low seroprevalence and low incidence of infection with toxoplasma gondii (nicolle et manceaux, 1908) in pediatric hematopoietic cell transplantation donors and recipients: Polish nationwide study. *Folia Parasitologica* 2019; **66**.

16.	Hakko E, Ozkan HA, Karaman K, Gulbas Z. Analysis of cerebral toxoplasmosis in a series of 170 allogeneic hematopoietic stem cell transplant patients. *Transplant Infectious Disease* 2013; **15**(6): 575–80.

17.	Chueng TA, Moroz IV, Anderson AD, Morris MI, Komanduri KV, Camargo JF. Failure of atovaquone prophylaxis for prevention of toxoplasmosis in hematopoietic cell transplant recipients.  Transplant Infectious Disease: Blackwell Publishing Inc.; 2020.

18.	Conrad A, Le Maréchal M, Dupont D, et al. A matched case–control study of toxoplasmosis after allogeneic haematopoietic stem cell transplantation: still a devastating complication. *Clinical Microbiology and Infection* 2016; **22**(7): 636–41.

19.	Mulanovich VE, Ahmed SI, Öztürk T, Khokhar FA, Kontoyiannis DP, De Lima M. Toxoplasmosis in allo-SCT patients: Risk factors and outcomes at a transplantation center with a low incidence. *Bone Marrow Transplantation* 2011; **46**(2): 273–7.

20.	Denier C, Bourhis JH, Lacroix C, et al. Spectrum and prognosis of neurologic complications after hematopoietic transplantation. *Neurology* 2006; **67**(11): 1990–7.

21.	Decembrino N, Comelli A, Genco F, et al. Toxoplasmosis disease in paediatric hematopoietic stem cell transplantation: Do not forget it still exists.  Bone Marrow Transplantation: Nature Publishing Group; 2017. p. 1326–9.

22.	Weber C, Schaper J, Tibussek D, et al. Diagnostic and therapeutic implications of neurological complications following paediatric haematopoietic stem cell transplantation. *Bone Marrow Transplant* 2008; **41**(3): 253–9.

23.	Aerts R, Mercier T, Beckers M, Schoemans H, Lagrou K, Maertens J. Toxoplasmosis after allogeneic haematopoietic cell transplantation: experience using a PCR-guided pre-emptive approach. *Clinical Microbiology and Infection* 2022; **28**(3): 440–5.

24.	Roemer E, Blau IW, Basara N, et al. Toxoplasmosis, a Severe Complication in Allogeneic Hematopoietic Stem Cell Transplantation: Successful Treatment Strategies during a 5-Year Single-Center Experience.

25.	Syed FI, Couriel DR, Frame D, Srinivasan A. Central Nervous System Complications of Hematopoietic Stem Cell Transplant. *Hematol Oncol Clin North Am* 2016; **30**(4): 887–98.

26.	Contopoulos-Ioannidis DG, Cho SM, Bertaina A, et al. Toxoplasmosis among 38751 hematopoietic stem-cell transplant recipients: A systematic review of disease prevalence and a compilation of imaging and autopsy findings.  Transplantation: Lippincott Williams and Wilkins; 2021. p. E375–E86.

27.	Castellano-Sanchez A, Popp AC, Nolte FS, et al. Acanthamoeba castellani encephalitis following partially mismatched related donor peripheral stem cell transplantation. *Transplant Infectious Disease* 2003; **5**(4): 191–4.

28.	Pemán J, Jarque I, Frasquet J, et al. Unexpected postmortem diagnosis of Acanthamoeba meningoencephalitis following allogeneic peripheral blood stem cell transplantation. *American journal of transplantation : official journal of the American Society of Transplantation and the American Society of Transplant Surgeons* 2008; **8**(7): 1562–6.

29.	Kaul DR, Lowe L, Visvesvara GS, Farmen S, Khaled YA, Yanik GA. Acanthamoeba infection in a patient with chronic graft-versus-host disease occurring during treatment with voriconazole. *Transplant Infectious Disease* 2008; **10**(6): 437–41.

30.	Keane NA, Lane LM, Canniff E, et al. A surviving case of acanthamoeba granulomatous amebic encephalitis in a hematopoietic stem cell transplant recipient. *American Journal of Case Reports* 2020; **21**: 1–6.

31.	Matsui T, Maeda T, Kusakabe S, et al. A case report of granulomatous amoebic encephalitis by Group 1 Acanthamoeba genotype T18 diagnosed by the combination of morphological examination and genetic analysis. *Diagnostic Pathology* 2018; **13**(1).

32.	Al Dhaheri F, Gunasekera N, Wardell H, Drobysheva A, Hussain S. Disseminated Acanthamoeba Infection in a Bone Marrow Transplant Recipient with CTLA-4 Haploinsufficiency.  Journal of Clinical Immunology: Springer; 2023. p. 319–22.

33.	Meconi F, Ciangola G, Mariotti B, et al. Cerebellar Neurocysticercosis as Long-Term Complication of Allogeneic Haematopoietic Stem Cell Transplantation from Haploidentical Donor. *Case Rep Infect Dis* 2019; **2019**: 4603130.

34.	Illade L, Molina Angulo B, Solís I, Diaz M, Gonzalez-Vicent M. Neurocysticercosis: An unusual seizure etiology in a hematopoietic stem cell transplanted patient. *Pediatr Hematol Oncol* 2018; **35**(1): 20–2.

35.	Purvey S, Lu K, Mukkamalla SK, et al. Conservative management of neurocysticercosis in a patient with hematopoietic stem cell transplantation: a case report and review. *Transpl Infect Dis* 2015; **17**(3): 456–62.

36.	White AC, Jr., Coyle CM, Rajshekhar V, et al. Diagnosis and Treatment of Neurocysticercosis: 2017 Clinical Practice Guidelines by the Infectious Diseases Society of America (IDSA) and the American Society of Tropical Medicine and Hygiene (ASTMH). *The American journal of tropical medicine and hygiene* 2018; **98**(4): 945–66.

37.	Maschke M, Dietrich U, Prumbaum M, et al. Opportunistic CNS infection after bone marrow transplantation. *Bone Marrow Transplant* 1999; **23**(11): 1167–76.

38.	Zaidman I, Shaziri T, Averbuch D, et al. Neurological complications following pediatric allogeneic hematopoietic stem cell transplantation: Risk factors and outcome. *Front Pediatr* 2022; **10**: 1064038.

39.	Sun YQ, Liu ZY, Huang XJ, et al. A Retrospective Study of Central Nervous System Invasive Fungal Disease after Allogeneic Stem Cell Transplantation: Risk Factors, Clinical Characteristics, and Outcomes. *Biol Blood Marrow Transplant* 2017; **23**(7): 1158–64.

40.	Zhang X, Zhang L, Li Y, Wang N, Zhang Y. Clinical performance of metagenomic next-generation sequencing for diagnosis of invasive fungal disease after hematopoietic cell transplant. *Front Cell Infect Microbiol* 2024; **14**: 1210857.

41.	Liu W, Fan Z, Zhang Y, et al. Metagenomic next-generation sequencing for identifying pathogens in central nervous system complications after allogeneic hematopoietic stem cell transplantation. *Bone Marrow Transplant* 2021; **56**(8): 1978–83.

42.	Glimåker M, Naucler P, Sjölin J. Etiology, clinical presentation, outcome and the effect of initial management in immunocompromised patients with community acquired bacterial meningitis. *J Infect* 2020; **80**(3): 291–7.

43.	van Veen KEB, Brouwer MC, van der Ende A, van de Beek D. Bacterial meningitis in hematopoietic stem cell transplant recipients: a population-based prospective study. *Bone Marrow Transplantation* 2016; **51**(11): 1490–5.

44.	Chung J, McCarthy KL, Redmond A, Butler J, Scott AP, Stewart AG. Listeria monocytogenes brain abscess as a late complication of allogeneic haemopoietic stem cell transplantation. *Intern Med J* 2021; **51**(6): 1005–6.

45.	Rivero GA, Torres HA, Rolston KV, Kontoyiannis DP. Listeria monocytogenes infection in patients with cancer. *Diagnostic microbiology and infectious disease* 2003; **47**(2): 393–8.

46.	Nolla-Salas J, Almela M, Coll P, Gasser I. Listeriosis in bone marrow transplant recipients. *Bone Marrow Transplant* 1997; **19**(9): 956–8.

47.	Safdar A, Papadopoulous EB, Armstrong D. Listeriosis in recipients of allogeneic blood and marrow transplantation: thirteen year review of disease characteristics, treatment outcomes and a new association with human cytomegalovirus infection. *Bone Marrow Transplant* 2002; **29**(11): 913–6.

48.	Chang J, Powles R, Mehta J, Paton N, Treleaven J, Jameson B. Listeriosis in bone marrow transplant recipients: incidence, clinical features, and treatment. *Clin Infect Dis* 1995; **21**(5): 1289–90.

49.	Vázquez E, de Gregorio O, Álvarez C, et al. Impact of immunosuppression on Listeria monocytogenes infection in Spain. *Pathogens and global health* 2025; **119**(5-6): 151–7.

50.	Averbuch D, Tridello G, Wendel L, et al. Listeria monocytogenes Infections in Hematopoietic Cell Transplantation Recipients: Clinical Manifestations and Risk Factors. A Multinational Retrospective Case-Control Study from the Infectious Diseases Working Party of the European Society for Blood and Marrow Transplantation. *Transplant Cell Ther* 2024; **30**(7): 712.e1–.e12.

51.	Charlier C, Perrodeau É, Leclercq A, et al. Clinical features and prognostic factors of listeriosis: the MONALISA national prospective cohort study. *The Lancet Infectious Diseases* 2017; **17**(5): 510–9.

52.	Zhang J, Mosunjac M, Moon A, Nooka A, Waller EK. Tuberculosis in umbilical cord blood transplant recipients: clinical characteristics and challenges. *Bone Marrow Transplant* 2015; **50**(3): 465–8.

53.	Zeng QZ, Zhang YY, Wu YJ, et al. Frequency, Risk Factors, and Outcome of Active Tuberculosis following Allogeneic Hematopoietic Stem Cell Transplantation. *Biol Blood Marrow Transplant* 2020; **26**(6): 1203–9.

54.	Maeda T, Kusumi E, Kami M, et al. Disseminated tuberculosis following reduced-intensity cord blood transplantation for adult patients with hematological diseases. *Bone Marrow Transplant* 2005; **35**(1): 91–7.

55.	Lee J, Lee MH, Kim WS, et al. Tuberculosis in hematopoietic stem cell transplant recipients in Korea. *Int J Hematol* 2004; **79**(2): 185–8.

56.	Lee HJ, Lee DG, Choi SM, et al. The demanding attention of tuberculosis in allogeneic hematopoietic stem cell transplantation recipients: High incidence compared with general population. *PLoS One* 2017; **12**(3): e0173250.

57.	Kmeid J, Kulkarni PA, Batista MV, et al. Active Mycobacterium tuberculosis infection at a comprehensive cancer center, 2006-2014. *BMC Infect Dis* 2019; **19**(1): 934.

58.	Khan B, Ahmed P, Ullah K, Hussain CA, Hussain I, Raza S. Frequency of tuberculosis in haematological malignancies and stem cell transplant recipients. *J Coll Physicians Surg Pak* 2005; **15**(1): 30–3.

59.	Russo RL, Dulley FL, Suganuma L, França IL, Yasuda MA, Costa SF. Tuberculosis in hematopoietic stem cell transplant patients: case report and review of the literature. *Int J Infect Dis* 2010; **14 Suppl 3**: e187–91.

60.	Drozd-Sokołowska J, Tridello G, Verheggen I, et al. Tuberculosis after hematopoietic cell transplantation: retrospective study on behalf of the Infectious Diseases Working Party of the EBMT. *Bone Marrow Transplant* 2025; **60**(5): 603–16.

61.	Zhang P, Huang L, Zheng M, et al. Case Report: Active tuberculosis infection in CAR T-cell recipients post CAR T-cell therapy: a retrospective case series. *Front Cell Infect Microbiol* 2023; **13**: 1147454.

62.	Schaller MA, Wicke F, Foerch C, Weidauer S. Central Nervous System Tuberculosis : Etiology, Clinical Manifestations and Neuroradiological Features. *Clin Neuroradiol* 2019; **29**(1): 3–18.

63.	Kontoyiannis DP, Marr KA, Park BJ, et al. Prospective surveillance for invasive fungal infections in hematopoietic stem cell transplant recipients, 2001-2006: overview of the Transplant-Associated Infection Surveillance Network (TRANSNET) Database. *Clin Infect Dis* 2010; **50**(8): 1091–100.

64.	Marr KA, Sun Y, Spec A, et al. A Multicenter, Longitudinal Cohort Study of Cryptococcosis in Human Immunodeficiency Virus-negative People in the United States. *Clin Infect Dis* 2020; **70**(2): 252–61.

65.	Rodríguez M, Chou S, Fisher DC, De Girolami U, Amato AA, Marty FM. Lyme meningoradiculitis and myositis after allogeneic hematopoietic stem cell transplantation. *Clin Infect Dis* 2005; **41**(12): e112–4.

66.	Schmidt-Hieber M, Ljungman P, Gilbert P, et al. Central nervous system disorders following haematopoietic stem cell transplantation: a prospective case&#x2013;control observational study from the Infectious Diseases Working Party and the Transplant Complications Working Party of EBMT. *eClinicalMedicine* 2026; **93**.

67.	Schmidt-Hieber M, Engelhard D, Ullmann A, et al. Central nervous system disorders after hematopoietic stem cell transplantation: a prospective study of the Infectious Diseases Working Party of EBMT. *J Neurol* 2020; **267**(2): 430–9.

68.	Mannina D, Berneking L, Both A, et al. Major central nervous system complications after allogeneic stem cell transplantation: A large retrospective study on 888 consecutive adult patients. *Eur J Haematol* 2020; **105**(6): 722–30.

69.	Dowling MR, Li S, Dey BR, et al. Neurologic complications after allogeneic hematopoietic stem cell transplantation: risk factors and impact. *Bone Marrow Transplant* 2018; **53**(2): 199–206.

70.	Hanajiri R, Kobayashi T, Yoshioka K, et al. Central nervous system infection following allogeneic hematopoietic stem cell transplantation. *Hematol Oncol Stem Cell Ther* 2017; **10**(1): 22–8.

71.	Balaguer Rosello A, Bataller L, Lorenzo I, et al. Infections of the Central Nervous System after Unrelated Donor Umbilical Cord Blood Transplantation or Human Leukocyte Antigen-Matched Sibling Transplantation. *Biol Blood Marrow Transplant* 2017; **23**(1): 134–9.

72.	Colombo AA, Marchioni E, Diamanti L, et al. Neurological Complications Involving the Central Nervous System After Allogeneic Hematopoietic Stem Cell Transplantation During a Period of Evolution in Transplant Modalities: A Cohort Analysis. *Transplantation* 2017; **101**(3): 616–23.

73.	Barba P, Piñana JL, Valcárcel D, et al. Early and late neurological complications after reduced-intensity conditioning allogeneic stem cell transplantation. *Biol Blood Marrow Transplant* 2009; **15**(11): 1439–46.

74.	Teive HA, Funke V, Bitencourt MA, et al. Neurological complications of hematopoietic stem cell transplantation (HSCT): a retrospective study in a HSCT center in Brazil. *Arq Neuropsiquiatr* 2008; **66**(3b): 685–90.

75.	Bjorklund A, Aschan J, Labopin M, et al. Risk factors for fatal infectious complications developing late after allogeneic stem cell transplantation. *Bone Marrow Transplant* 2007; **40**(11): 1055–62.

76.	Uckan D, Cetin M, Yigitkanli I, et al. Life-threatening neurological complications after bone marrow transplantation in children. *Bone Marrow Transplant* 2005; **35**(1): 71–6.

77.	Faraci M, Lanino E, Dini G, et al. Severe neurologic complications after hematopoietic stem cell transplantation in children. *Neurology* 2002; **59**(12): 1895–904.

78.	Oyama T, Kageyama K, Araoka H, et al. Clinical and microbiological characteristics of bacterial meningitis in umbilical cord blood transplantation recipients. *Int J Hematol* 2022; **116**(6): 966–72.

79.	Okinaka K, Inoue Y, Uchida N, et al. Clinical characteristics and risk factors of pneumococcal diseases in recipients of allogeneic hematopoietic stem cell transplants in the late phase: A retrospective registry study. *J Infect Chemother* 2023; **29**(7): 726–30.

80.	Olarte L, Lin PL, Barson WJ, et al. Invasive pneumococcal infections in children following transplantation in the pneumococcal conjugate vaccine era. *Transpl Infect Dis* 2017; **19**(1).

81.	Torda A, Chong Q, Lee A, et al. Invasive pneumococcal disease following adult allogeneic hematopoietic stem cell transplantation. *Transpl Infect Dis* 2014; **16**(5): 751–9.

82.	Debbache K, Varon E, Hicheri Y, et al. The epidemiology of invasive Streptococcus pneumoniae infections in onco-haematology and haematopoietic stem cell transplant patients in France. Are the serotypes covered by the available anti-pneumococcal vaccines? *Clin Microbiol Infect* 2009; **15**(9): 865–8.

83.	Kumar D, Humar A, Plevneshi A, et al. Invasive pneumococcal disease in adult hematopoietic stem cell transplant recipients: a decade of prospective population-based surveillance. *Bone Marrow Transplant* 2008; **41**(8): 743–7.

84.	Engelhard D, Cordonnier C, Shaw PJ, et al. Early and late invasive pneumococcal infection following stem cell transplantation: a European Bone Marrow Transplantation survey. *Br J Haematol* 2002; **117**(2): 444–50.

85.	Karaman S, Kebudi R, Kizilocak H, et al. Central Nervous System Fungal Infections in Children With Leukemia and Undergoing Hematopoietic Stem Cell Transplantation: A Retrospective Multicenter Study. *J Pediatr Hematol Oncol* 2022; **44**(8): e1039–e45.

86.	Candoni A, Facchin G, Busca A, et al. Central nervous system fungal infections in allogeneic stem cell transplantation. Outcome of 24 recent cases and literature review. *Eur J Haematol* 2020; **104**(2): 148–50.

87.	Lauten M, Attarbaschi A, Cario G, et al. Invasive mold disease of the central nervous system in children and adolescents with cancer or undergoing hematopoietic stem cell transplantation: Analysis of 29 contemporary patients. *Pediatr Blood Cancer* 2019; **66**(8): e27806.

88.	Economides MP, Ballester LY, Kumar VA, et al. Invasive mold infections of the central nervous system in patients with hematologic cancer or stem cell transplantation (2000-2016): Uncommon, with improved survival but still deadly often. *J Infect* 2017; **75**(6): 572–80.

89.	Balaguer Rosello A, Bataller L, Lorenzo I, et al. Infections of the Central Nervous System after Unrelated Donor Umbilical Cord Blood Transplantation or Human Leukocyte Antigen-Matched Sibling Transplantation. *Biol Blood Marrow Transplant* 2017; **23**(1): 134–9.

90.	Jantunen E, Volin L, Salonen O, et al. Central nervous system aspergillosis in allogeneic stem cell transplant recipients. *Bone Marrow Transplant* 2003; **31**(3): 191–6.

91.	Baddley JW, Salzman D, Pappas PG. Fungal brain abscess in transplant recipients: epidemiologic, microbiologic, and clinical features. *Clin Transplant* 2002; **16**(6): 419–24.

92.	Donnelly JP, Chen SC, Kauffman CA, et al. Revision and Update of the Consensus Definitions of Invasive Fungal Disease From the European Organization for Research and Treatment of Cancer and the Mycoses Study Group Education and Research Consortium. *Clin Infect Dis* 2020; **71**(6): 1367–76.

93.	De Pauw B, Walsh TJ, Donnelly JP, et al. Revised definitions of invasive fungal disease from the European Organization for Research and Treatment of Cancer/Invasive Fungal Infections Cooperative Group and the National Institute of Allergy and Infectious Diseases Mycoses Study Group (EORTC/MSG) Consensus Group. *Clin Infect Dis* 2008; **46**(12): 1813–21.

94.	Malek AE, Al-Juhaishi T, Milton DR, et al. Outcomes of toxoplasmosis after allogeneic hematopoietic stem cell transplantation and the role of antimicrobial prophylaxis. *Bone Marrow Transplant* 2024; **59**(5): 699–704.

95.	Xhaard A, Villate A, Hamane S, et al. A 10-year retrospective analysis of Toxoplasma gondii qPCR screening in allogeneic hematopoietic stem cell transplantation recipients. *Bone Marrow Transplant* 2023; **58**(2): 152–9.

96.	Aerts R, Mercier T, Beckers M, Schoemans H, Lagrou K, Maertens J. Toxoplasmosis after allogeneic haematopoietic cell transplantation: experience using a PCR-guided pre-emptive approach. *Clin Microbiol Infect* 2022; **28**(3): 440–5.

97.	Paccoud O, Guitard J, Labopin M, et al. Features of Toxoplasma gondii reactivation after allogeneic hematopoietic stem-cell transplantation in a high seroprevalence setting. *Bone Marrow Transplant* 2020; **55**(1): 93–9.

98.	Czyzewski K, Fraczkiewicz J, Salamonowicz M, et al. Low seroprevalence and low incidence of infection with Toxoplasma gondii (Nicolle et Manceaux, 1908) in pediatric hematopoietic cell transplantation donors and recipients: Polish nationwide study. *Folia Parasitol* 2019; **66**.

99.	Chueng TA, Moroz IV, Anderson AD, Morris MI, Komanduri KV, Camargo JF. Failure of atovaquone prophylaxis for prevention of toxoplasmosis in hematopoietic cell transplant recipients. *Transpl Infect Dis* 2020; **22**(1): e13198.

100.	Robin C, Leclerc M, Angebault C, et al. Toxoplasmosis as an Early Complication of Allogeneic Hematopoietic Cell Transplantation. *Biol Blood Marrow Transplant* 2019; **25**(12): 2510–3.

101.	Decembrino N, Comelli A, Genco F, et al. Toxoplasmosis disease in paediatric hematopoietic stem cell transplantation: do not forget it still exists. *Bone Marrow Transplant* 2017; **52**(9): 1326–9.

102.	Conrad A, Le Maréchal M, Dupont D, et al. A matched case-control study of toxoplasmosis after allogeneic haematopoietic stem cell transplantation: still a devastating complication. *Clin Microbiol Infect* 2016; **22**(7): 636–41.

103.	Mendorf A, Klyuchnikov E, Langebrake C, et al. Atovaquone for Prophylaxis of Toxoplasmosis after Allogeneic Hematopoietic Stem Cell Transplantation. *Acta Haematol* 2015; **134**(3): 146–54.

104.	Hakko E, Ozkan HA, Karaman K, Gulbas Z. Analysis of cerebral toxoplasmosis in a series of 170 allogeneic hematopoietic stem cell transplant patients. *Transpl Infect Dis* 2013; **15**(6): 575–80.

105.	Sumi M, Aosai F, Norose K, et al. Acute exacerbation of Toxoplasma gondii infection after hematopoietic stem cell transplantation: five case reports among 279 recipients. *Int J Hematol* 2013; **98**(2): 214–22.

106.	Mulanovich VE, Ahmed SI, Öztürk T, Khokhar FA, Kontoyiannis DP, de Lima M. Toxoplasmosis in allo-SCT patients: risk factors and outcomes at a transplantation center with a low incidence. *Bone Marrow Transplant* 2011; **46**(2): 273–7.

107.	Meers S, Lagrou K, Theunissen K, et al. Myeloablative conditioning predisposes patients for Toxoplasma gondii reactivation after allogeneic stem cell transplantation. *Clin Infect Dis* 2010; **50**(8): 1127–34.

108.	Roemer E, Blau IW, Basara N, et al. Toxoplasmosis, a severe complication in allogeneic hematopoietic stem cell transplantation: successful treatment strategies during a 5-year single-center experience. *Clin Infect Dis* 2001; **32**(1): E1–8.

109.	Martino R, Bretagne S, Einsele H, et al. Early detection of Toxoplasma infection by molecular monitoring of Toxoplasma gondii in peripheral blood samples after allogeneic stem cell transplantation. *Clin Infect Dis* 2005; **40**(1): 67–78.

110.	Al Dhaheri F, Gunasekera N, Wardell H, Drobysheva A, Hussain S. Disseminated Acanthamoeba Infection in a Bone Marrow Transplant Recipient with CTLA-4 Haploinsufficiency. *J Clin Immunol* 2023; **43**(2): 319–22.

111.	Keane NA, Lane LM, Canniff E, et al. A Surviving Case of Acanthamoeba Granulomatous Amebic Encephalitis in a Hematopoietic Stem Cell Transplant Recipient. *Am J Case Rep* 2020; **21**: e923219.

112.	Matsui T, Maeda T, Kusakabe S, et al. A case report of granulomatous amoebic encephalitis by Group 1 Acanthamoeba genotype T18 diagnosed by the combination of morphological examination and genetic analysis. *Diagn Pathol* 2018; **13**(1): 27.

113.	Coven SL, Song E, Steward S, et al. Acanthamoeba granulomatous amoebic encephalitis after pediatric hematopoietic stem cell transplant. *Pediatr Transplant* 2017; **21**(8).

114.	Doan N, Rozansky G, Nguyen HS, et al. Granulomatous amebic encephalitis following hematopoietic stem cell transplantation. *Surg Neurol Int* 2015; **6**(Suppl 18): S459–62.

115.	Kaul DR, Lowe L, Visvesvara GS, Farmen S, Khaled YA, Yanik GA. Acanthamoeba infection in a patient with chronic graft-versus-host disease occurring during treatment with voriconazole. *Transpl Infect Dis* 2008; **10**(6): 437–41.

116.	Castellano-Sanchez A, Popp AC, Nolte FS, et al. Acanthamoeba castellani encephalitis following partially mismatched related donor peripheral stem cell transplantation. *Transpl Infect Dis* 2003; **5**(4): 191–4.

117.	Haddad N, Carr M, Balian S, et al. The Blood-Brain Barrier and Pharmacokinetic/Pharmacodynamic Optimization of Antibiotics for the Treatment of Central Nervous System Infections in Adults. *Antibiotics (Basel)* 2022; **11**(12).

118.	Bodilsen J, D'Alessandris QG, Humphreys H, et al. European society of Clinical Microbiology and Infectious Diseases guidelines on diagnosis and treatment of brain abscess in children and adults. *Clin Microbiol Infect* 2024; **30**(1): 66–89.

119.	Patterson TF, Thompson GR, 3rd, Denning DW, et al. Practice Guidelines for the Diagnosis and Management of Aspergillosis: 2016 Update by the Infectious Diseases Society of America. *Clin Infect Dis* 2016; **63**(4): e1–e60.

120.	Dadwal SS, Hohl TM, Fisher CE, et al. American Society of Transplantation and Cellular Therapy Series, 2: Management and Prevention of Aspergillosis in Hematopoietic Cell Transplantation Recipients. *Transplant Cell Ther* 2021; **27**(3): 201–11.

121.	Ullmann AJ, Aguado JM, Arikan-Akdagli S, et al. Diagnosis and management of Aspergillus diseases: executive summary of the 2017 ESCMID-ECMM-ERS guideline. *Clin Microbiol Infect* 2018; **24 Suppl 1**: e1–e38.

122.	Lamoth F, Mercier T, Andre P, et al. Isavuconazole brain penetration in cerebral aspergillosis. *J Antimicrob Chemother* 2019; **74**(6): 1751–3.

123.	Schwartz S, Cornely OA, Hamed K, et al. Isavuconazole for the treatment of patients with invasive fungal diseases involving the central nervous system. *Med Mycol* 2020; **58**(4): 417–24.

124.	Schwartz S, Ruhnke M, Ribaud P, et al. Improved outcome in central nervous system aspergillosis, using voriconazole treatment. *Blood* 2005; **106**(8): 2641–5.

125.	Lanternier F, Poiree S, Elie C, et al. Prospective pilot study of high-dose (10 mg/kg/day) liposomal amphotericin B (L-AMB) for the initial treatment of mucormycosis. *J Antimicrob Chemother* 2015; **70**(11): 3116–23.

126.	Cornely OA, Alastruey-Izquierdo A, Arenz D, et al. Global guideline for the diagnosis and management of mucormycosis: an initiative of the European Confederation of Medical Mycology in cooperation with the Mycoses Study Group Education and Research Consortium. *Lancet Infect Dis* 2019; **19**(12): e405–e21.

127.	Pitisuttithum P, Negroni R, Graybill JR, et al. Activity of posaconazole in the treatment of central nervous system fungal infections. *J Antimicrob Chemother* 2005; **56**(4): 745–55.

128.	Kontoyiannis DP, Lewis RE. How I treat mucormycosis. *Blood* 2011; **118**(5): 1216–24.

129.	Cornely OA, Maertens J, Bresnik M, et al. Liposomal amphotericin B as initial therapy for invasive mold infection: a randomized trial comparing a high-loading dose regimen with standard dosing (AmBiLoad trial). *Clin Infect Dis* 2007; **44**(10): 1289–97.

130.	Maertens J, Birne R, Felton T, Neofytos D, Hoenigl M. Liposomal amphotericin B and renal safety: review of the evidence and clinical considerations. *J Antimicrob Chemother* 2026; **81**(2).

131.	Margalit I, Lebeaux D, Tishler O, et al. How do I manage nocardiosis? *Clin Microbiol Infect* 2021; **27**(4): 550–8.

132.	Coussement J, Lebeaux D, Rouzaud C, Lortholary O. Nocardia infections in solid organ and hematopoietic stem cell transplant recipients. *Current opinion in infectious diseases* 2017; **30**(6): 545–51.

133.	Meena DS, Kumar D, Bohra GK, Midha N, Garg MK. Clinical Characteristics and Treatment Outcome of Central Nervous System Nocardiosis: A Systematic Review of Reported Cases. *Medical principles and practice : international journal of the Kuwait University, Health Science Centre* 2022; **31**(4): 333–41.

134.	Sutter JP, Kocheise L, Kempski J, et al. Gentamicin combination treatment is associated with lower mortality in patients with invasive listeriosis: a retrospective analysis. *Infection* 2024; **52**(4): 1601–6.

135.	Arslan F, Meynet E, Sunbul M, et al. The clinical features, diagnosis, treatment, and prognosis of neuroinvasive listeriosis: a multinational study. *Eur J Clin Microbiol Infect Dis* 2015; **34**(6): 1213–21.

136.	Dickstein Y, Oster Y, Shimon O, et al. Antibiotic treatment for invasive nonpregnancy-associated listeriosis and mortality: a retrospective cohort study. *Eur J Clin Microbiol Infect Dis* 2019; **38**(12): 2243–51.

137.	Fernández Guerrero ML, Torres R, Mancebo B, et al. Antimicrobial treatment of invasive non-perinatal human listeriosis and the impact of the underlying disease on prognosis. *Clin Microbiol Infect* 2012; **18**(7): 690–5.

138.	Brouwer MC, van de Beek D. Adjunctive dexamethasone treatment in adults with listeria monocytogenes meningitis: a prospective nationwide cohort study. *EClinicalMedicine* 2023; **58**: 101922.

139.	Pelegrín I, Moragas M, Suárez C, et al. Listeria monocytogenes meningoencephalitis in adults: analysis of factors related to unfavourable outcome. *Infection* 2014; **42**(5): 817–27.

140.	Amaya-Villar R, García-Cabrera E, Sulleiro-Igual E, et al. Three-year multicenter surveillance of community-acquired Listeria monocytogenes meningitis in adults. *BMC Infect Dis* 2010; **10**: 324.

141.	Mitjà O, Pigrau C, Ruiz I, et al. Predictors of mortality and impact of aminoglycosides on outcome in listeriosis in a retrospective cohort study. *J Antimicrob Chemother* 2009; **64**(2): 416–23.

142.	van de Beek D, Cabellos C, Dzupova O, et al. ESCMID guideline: diagnosis and treatment of acute bacterial meningitis. *Clin Microbiol Infect* 2016; **22 Suppl 3**: S37–62.
